# Targeting glioblastoma with NK cells and mAb against NG2/CSPG4 prolongs animal survival

**DOI:** 10.18632/oncotarget.1291

**Published:** 2013-09-09

**Authors:** Aurélie Poli, Jian Wang, Olivia Domingues, Jesús Planagumà, Tao Yan, Cecilie Brekke Rygh, Kai Ove Skaftnesmo, Frits Thorsen, Emmet McCormack, François Hentges, Paal Henning Pedersen, Jacques Zimmer, Per Øyvind Enger, Martha Chekenya

**Affiliations:** ^1^ Translational Cancer Research, Department of Biomedicine, University of Bergen, Norway; ^2^ Laboratoire d'Immunogénétique-Allergologie, CRP-Santé, Luxembourg; ^3^ Department of Medicine, Haukeland University Hospital, Bergen, Norway; ^4^ Department of Neurosurgery, Haukeland University Hospital, Bergen, Norway; ^5^ Department of Clinical Dentistry, University of Bergen, Norway; ^6^ Department of Neurosurgery, Qilu Hospital, Shandong University, P.R. China

**Keywords:** Microglia, NK cells, glioblastoma, immunotherapy, NG2/CSPG4

## Abstract

Glioblastoma (GBM) is the most malignant brain tumor where patients' survival is only 14.6 months, despite multimodal therapy with debulking surgery, concurrent chemotherapy and radiotherapy. There is an urgent, unmet need for novel, effective therapeutic strategies for this devastating disease. Although several immunotherapies are under development for the treatment of GBM patients, the use of natural killer (NK) cells is still marginal despite this being a promising approach to treat cancer. In regard of our knowledge on the role of NG2/*CSPG4* in promoting GBM aggressiveness we investigated the potential of an innovative immunotherapeutic strategy combining mAb9.2.27 against NG2/*CSPG4* and NK cells in preclinical animal models of GBM. Multiple immune escape mechanisms maintain the tumor microenvironment in an anti-inflammatory state to promote tumor growth, however, the distinct roles of resident microglia *versus* recruited macrophages is not elucidated. We hypothesized that exploiting the cytokine release capabilities of activated NK cells to reverse the anti-inflammatory axis combined with mAb9.2.27 targeting the NG2/*CSPG4* may favor tumor destruction by editing pro-GBM immune responses. Combination treatment with NK+mAb9.2.27 diminished tumor growth that was associated with reduced tumor proliferation, increased cellular apoptosis and prolonged survival compared to vehicle and monotherapy controls. The therapeutic efficacy was mediated by recruitment of CCR2^low^ macrophages into the tumor microenvironment, increased ED1 and MHC class II expression on microglia that might render them competent for GBM antigen presentation, as well as elevated IFN-γ and TNF-α levels in the cerebrospinal fluid compared to controls. Depletion of systemic macrophages by liposome-encapsulated clodronate decreased the CCR2^low^ macrophages recruited to the brain and abolished the beneficial outcomes. Moreover, mAb9.2.27 reversed tumor-promoting effects of patient-derived tumor-associated macrophage/ microglia (TAM) *ex vivo*. Taken together, these findings indicate that NK+mAb9.2.27 treatment may be an amenable therapeutic strategy to treat NG2/*CSPG4* expressing GBMs. We provide a novel conceptual approach of combination immunotherapy for glioblastoma. The results traverse beyond the elucidation of NG2/*CSPG4* as a therapeutic target, but demonstrate a proof of concept that this antibody may hold potential for the treatment of GBM by activation of tumor infiltrated microglia/macrophages.

## INTRODUCTION

Glioblastoma (GBM) is the most common and malignant brain tumor in adults, classified as grade IV astrocytoma by the World Health Organization (WHO) [1]. Despite the multimodal treatment consisting of debulking surgery, radiotherapy and chemotherapy, the prognosis remains dismal, with a median survival of 14.6 months [2]. The main challenge for successfully treating GBM is its diffuse invasion of the brain parenchyma that renders the tumor cells refractory to surgery, chemo-radiotherapy and immune surveillance, leading invariably to recurrence. Moreover, the molecular and cellular heterogeneity of GBM underlies their inherent resistance to radio- and chemotherapy. Thus, there is an urgent need for identifying cancer biomarkers that drive malignant progression and that may be amenable for effective combination immunotherapeutic approaches. Indeed there is a growing interest in establishing novel immunotherapeutic approaches to the management of GBM patients. This is boosted partly by the Federal Drug Administrations (FDA) approval of Sipuleucel-T for prostate cancer and Ipilimumab for metastatic melanoma treatment. Moreover, the continual discovery of novel tumor antigens that are abundantly and specifically expressed in GBM tissue compared to normal brain [3, 4]may spur further interest in vaccine based immunotherapies.

Potential targets may also be the interactions between tumor cells and constituents of the microenvironment that regulate the bioavailability of ligands for signaling receptors and promotion of malignant progression. Amongst these, proteoglycans could impact on malignant progression by binding growth factors, sequestration of chemokines and proteases. Recently the knockdown of the SULF2 heparin sulfatase in astrocytoma was demonstrated to the diminish activity of several receptor tyrosine kinase (RTK) signaling pathways known to be active in GBM[5]. Our team previously demonstrated the existence of a subpopulation of GBM cells expressing Neuroglial-2 (NG2/*CSPG4*) transmembrane chondroitin sulfate proteoglycan, with an immature phenotype denoted by nestin and vimentin positivity, but not CD133 [6]. The gene, *CSPG4*, encoding the NG2 or human melanoma proteoglycan, is turned off upon terminal differentiation, but is aberrantly re-expressed by several tumor types [7-10]. Recently, we described that 50 % of GBM patients' biopsies show high levels of NG2/*CSPG4* expression and that this was an independent prognostic factor for shorter patient survival [6]. These NG2/*CSPG4* positive GBMs also corresponded to the proliferative and mesenchymal molecular phenotypes that are associated with poor prognosis [11]. In addition, we demonstrated that NG2/*CSPG4* expression by GBM cells promotes angiogenesis [8], cellular proliferation [12], and chemo-resistance [13]. In this latter study, we demonstrated that NG2/*CSPG4* expressing GBM cells were highly resistance to tumor necrosis factor alpha (TNF-α) mediated apoptosis due to elevated PI3K/Akt survival signaling. NG2/*CSPG4*'s impact on the key hallmarks of GBM and its cell surface expression on both tumor and neovasculature render it an amenable target for immunotherapy using monoclonal antibodies that aid the recognition and destruction of malignant cells via the immune system. The adoptive cell transfer (ACT) of autologous or allogeneic lymphocytes to treat cancer has demonstrated great therapeutic potential in early clinical trials of several solid malignancies, including melanoma and neuroblastoma [14, 15]. A number of phase I/II clinical trials have been conducted using ACT of lymphokine activated killer (LAK) cells or cytotoxic T lymphocytes (CTLs) to treat GBM. To date the efficacy of adoptive cellular therapies has been moderate, partly due to the need to generate tumor specific lymphocytes for each individual patient, which poses technical and economic hurdles for fast tracking the treatments to the clinic. To bypass these limitations Marcus et al., [14] pioneered chimeric antigen receptor (CAR) redirected T cells that are composed of single chain antibody fragments fused to T cell activating signaling motifs. This endows the allogeneic T cells major histocompatibility complex (MHC) unrestricted tumor specificity and thus, could be used as “off the shelf, universal effector cells” against cancer in all patients. However, ACT of allogeneic T cells poses the inherent risk of host-*versus*-graft response (HvG) and the danger of graft-*versus*-host disease (HvHD).

Among cytotoxic lymphocytes, NK cells are the most efficient effectors against tumors, capable of direct killing without prior immunization [16]. So far only two studies investigating purified NK cells for GBM treatment have been conducted, and they demonstrated a preferential killing of GBM stem-like cells [17, 18] purported to contribute to therapy resistance and recurrence. Several studies have demonstrated that NK cells can modulate the development of tumor-specific cytotoxic T lymphocytes and induce a T-helper 1 (Th1) cytokine profile in cancer [19-21]. Moreover, the immunomodulation capacity of NK cells through cytokine secretion could skew tumor-associated macrophages (TAM) from anti-inflammatory to pro-inflammatory phenotypes [22] and was shown to enhance tumor immunoediting [23]. These last properties are essential for the success of an immunotherapy against GBM where up to 70 % of the tumor mass represent TAM polarized to the M2 phenotype that promote tumor cell proliferation [24], [25]. While NK cell therapies were shown to be effective in hematological malignancies, there is a paucity of studies evaluating their efficacy in brain tumors *in vivo* [26]. Whereas most studies utilize NK cells for their direct cytotoxicity capabilities, in the present study we investigated a novel approach to exploit the potential of NK cells to revert the immune contexture from anti-inflammatory to pro-inflammatory through cytokine release. We further investigated the therapeutic potential of NK cells to induce antibody dependent cellular cytotoxicity (ADCC) in the brain through ligation of the mAb9.2.27 directed against NG2/*CSPG4*. The mAb9.2.27 is one of the first monoclonals ever to be produced against NG2/*CSPG4*, as well as one of the antibodies having the longest track-record of being exploited to target NG2/*CSPG4*-positive cancer cells *in vitro* and *in vivo*. All attempts to use mAb9.2.27 to abrogate tumors *in vivo* have focused upon the use of antibody complexes carrying cytotoxic agents, whereas no published study has clearly demonstrated a direct anti-neoplastic effect of the naked antibody. Other anti-NG2/*CSPG4* antibodies have been reported to display an anti-tumor potential *in vivo*, but none of these have proven to be effective on glioblastoma and none have been extensively tested in combination therapy with immune cells. Thus, the present study provides a novel conceptual approach to the combination immunotherapeutic treatment of glioblastoma. Our findings have greater implications beyond the elucidation of NG2/*CSPG4* as a therapeutic target, but demonstrate a proof of concept that mAb9.2,27 could activate cytotoxic functions of glioma infiltrated microglia/macrophages that may further hold therapeutic potential.

The principal aims of the present paper were to investigate the therapeutic efficacy of combining adoptively transferred, purified, activated NK cells with passive immunotherapy using mAb9.2.27 in GBM-bearing rats and to identify the mechanisms and cellular subsets mediating the anti-tumor effects. We demonstrated that the combination treatment with activated NK cells and mAb9.2.27 eradicated the tumor more efficiently compared to monotherapies with mAb9.2.27 or NK cells and vehicle-treated controls. The mechanism involved the recruitment of macrophages/microglia with a pro-inflammatory phenotype into the tumor. In addition, activated macrophages/microglia became highly cytotoxic against tumor cells *ex vivo* in presence of mAb9.2.27

## RESULTS

### Combination treatment with mAb9.2.27 and adoptively transferred NK cells diminishes GBM cell proliferation and increases survival

**Figure 1:**
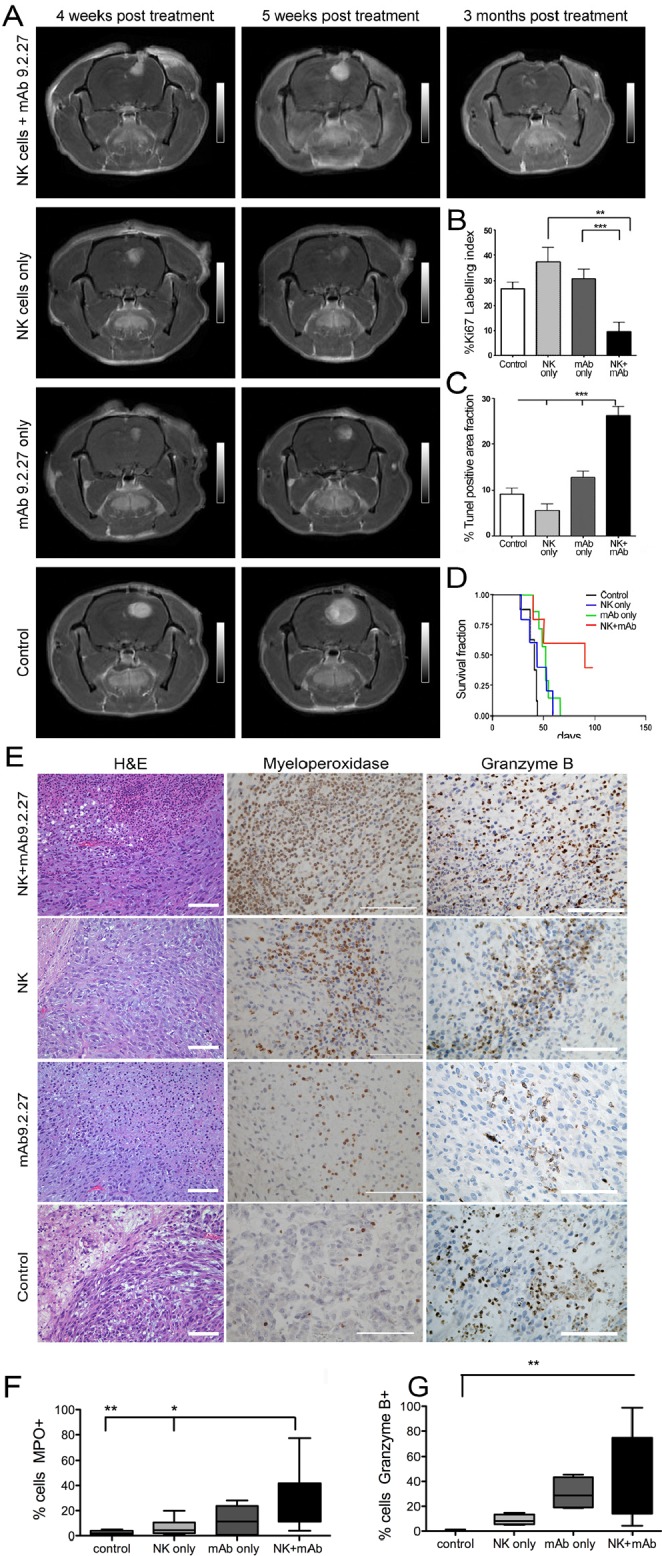
Combination NK+mAb9.2.27 treatment ablates tumor growth, prolongs survival and presented a tropism for cytotoxic cells in combination treated in U87MG bearing rats (A) Longitudinal axial post-contrast T1-weighted images of nude rats bearing U87MG tumors treated with combination NK+mAb9.2.27, NK cell monotherapy, mAb9.2.27 monotherapy, and untreated controls, showing the same animal after 4, 5 weeks and 3 months post NK+mAb9.2.27 treatment. (B), (C) Plot showing the quantification of (B) tumor proliferation by % Ki67 labeling index and (C) cell death by % area fraction TUNEL positive apoptotic/necrotic cells. Data represent mean ± SEM of all tumors in the groups. ***p<0.001 and **p<0.01. (D) Kaplan –Meier survival curves showing surviving fraction, n=7 animals/group. (E) H&E staining of U87MG tumor with necrosis (scale bar 200μm, magnification 100X). Increased myeloperoxidase positive cells in mAb9.2.27 and combination treated tumors compared to control and NK treated tumors (scale bar 100μm, magnification 200X). High granzyme B positivity in combination treated tumors (scale bar 100μm, magnification 400X). Quantification of (F) MPO and (G) granzyme B positive cells. Data in (F) and (G) represent mean SEM from all tumors in the groups. *p<0.05, **p<0.001.

We demonstrated previously that elevated levels of the NG2/*CSPG4* proteoglycan on GBM cells and angiogenic vasculature is associated with a more aggressive disease course [6, 8, 12, 13]. We therefore hypothesized that perturbation of NG2/*CSPG4* signaling with mAb9.2.27 alone or in combination with adoptively transferred NK cells might have therapeutic benefits for tumor-bearing rats. First we investigated the efficacy of the combination treatment in eradicating U87MG gliomas that are 99.2±0.2 % (n=3) NG2/*CSPG4* positive, as recognized by mAb9.2.27 (Supplementary Fig. 1A). Four weeks after treatment, control untreated U87MG tumors were strongly contrast enhancing on T1-weighted MR images indicating increased angiogenesis and rapid growth compared to monotherapy and combination treated animals (Fig. 1A). However, while the monotherapy groups exhibited initial radiological responses of reduced tumor sizes on T1 weighted MRI with contrast, (Fig. 1A), after 5 weeks both monotherapy and control tumors progressed and killed their hosts. The NK+mAb9.2.27 combination treated tumors regressed as indicated by dramatically diminished contrast enhancement in MR images 3 months post-treatment (Fig. 1A). Tumor cell proliferation was significantly attenuated in the combination treatment compared to all other groups (One way ANOVA F_7.4_, NK p=0.006, n=6; mAb9.2.27 and control p=0.001, n=5), (Fig. 1B). The tumors treated with combined NK+mAb9.2.27 contained significantly larger areas with apoptotic and necrotic tissue compared to all other treatments (One way ANOVA F_20_, df=3, p=0.0001, n=32), (Fig. 1C). Correspondingly, the combined treatment significantly prolonged the survival of the animals with a median survival time of 91 days compared to 52, 44, and 39 days in the mAb9.2.27, NK cell and control groups, respectively (Log Rank _9.3_, df=3, p=0.026, n=7), (Fig. 1D). In NK+mAb9.2.27 group, 60 % of the animals sacrificed for autopsy displayed necrosis with no visible tumor mass.

Next, we investigated the requirement for NG2/*CSPG4* recognition in NK+mAb9.2.27 therapeutic efficacy in U251-NG2 GBM tumors that stably overexpress rat NG2/*CSPG4* but have a lower affinity for the human mAb9.2.27 as indicated by 34.5±4.2 % (n=3) positivity, (Supplementary Fig. 1B). T1-weighted MR images showed contrast agent leakage in all tumors one week prior to treatment. Treatment with mAb9.2.27 alone or in combination with NK cells diminished contrast agent leakage at 10 days post treatment compared to control tumors. However, all the tumors continued to grow as seen on T1-weighted MRI after 3 weeks (Supplementary Fig. 2A). Optical imaging demonstrated the presence of mAb9.2.27 retained in the tumor bed 5 days post-infusion while the IgG2a isotype control antibodies could not be visualized (Supplementary Fig. 2B), indicating that the mAb9.2.27 was stable within the tumor for at least 5 days. In contrast to the U87MG tumors, Ki67 labeling index revealed increased proliferation of the NK+mAb9.2.27 combination treated tumors, compared to control (ANOVA F_14.24_, df=3, p=0.0001, n=5), mAb9.2.27 treated tumors (p=0.0001, n=7) and NK cell treated tumors (p=0.001, n=6), (Supplementary Fig. 2C). However, the combination treated tumors also contained greater areas with necrotic/apoptotic cells compared to control and monotherapy groups (ANOVA F_2.8_, df=3, p=0.0001, n=5), (Supplementary Fig. 2D). The latter finding was reflected in the survival curves, as the NK+mAb9.2.27 combination treatment marginally prolonged the median survival to 34.5 days compared to 24 days in NK cell monotherapy (p=0.0018, n=6), 32 days in mAb9.2.27 monotherapy (p=0.014, n=5) and 29 days in the vehicle-treated controls, (p=0.0011, n=4) at the termination of the experiment. Thus, the difference in therapeutic efficacy measured as median survival of the animals treated with NK+mAb9.2.27 in the U87MG (91 days) *versus* U251-NG2 (34.5 days) tumors might be due to the specificity of NG2/*CSPG4* recognition by mAb9.2.27.

### Differential recruitment of activated immune cells expressing cytotoxic granules regulates the therapeutic response in NK+mAb9.2.27 combination therapy

Histological analyses of U87MG tumors revealed that while untreated control and NK cell monotherapy treated tumors were angiogenic with no discernible central necrosis, NK+mAb9.2.27 and mAb9.2.27 treated tumors exhibited large central necrosis, densely filled with leucocytes (Fig. 1E). The necrotic areas showed an intense myeloperoxidase (MPO) activity in the combination treated tumors, whereas it was markedly attenuated in NK cell monotherapy and control groups (One-way ANOVA _14.97_, df=3, p=0.0018, n=5), (Fig. 1E and 1F). Granzyme B was also abundantly expressed after NK+mAb9.2.27 treatment in areas adjacent to focal necrosis/apoptosis compared to the control tumors (One way ANOVA _13.21_, df=3, p=0.0042, n=4), (Fig. 1E and 1G). Similar to U87MG tumors, histological analyses of U251-NG2 tumors also revealed that both mAb9.2.27 and NK+mAb9.2.27 treated tumors exhibited extensive tissue necrosis, packed with leucocytes (supplementary Fig. 3A), and more abundant MPO expressing cells were present in the NK+mAb9.2.27 treated tumors compared to NK cell and mAb9.2.27 monotherapy, as well as and control tumours (One-way ANOVA _4.07_, df=3, p<0.0001, n=5), (supplementary Fig. 3A and 3B). Granzyme B was abundantly expressed in the mAb9.2.27 treated tumors compared to NK cell monotherapy and controls in areas adjacent to focal necrosis/apoptosis (One-Way ANOVA _19.90_ df=3, p=0.0002, n=5), (Supplementary Fig. 3A and 3C). Interferon-γ was only expressed in the mAb9.2.27 and NK+mAb9.2.27 combination treated tumors (supplementary Fig. 3A). Collectively, these data indicate a tropism for activated immune cells with the capacity to secrete cytotoxic granules within tumor in the mAb9.2.27 and NK+mAb9.2.27 treated rats.

**Figure 2:**
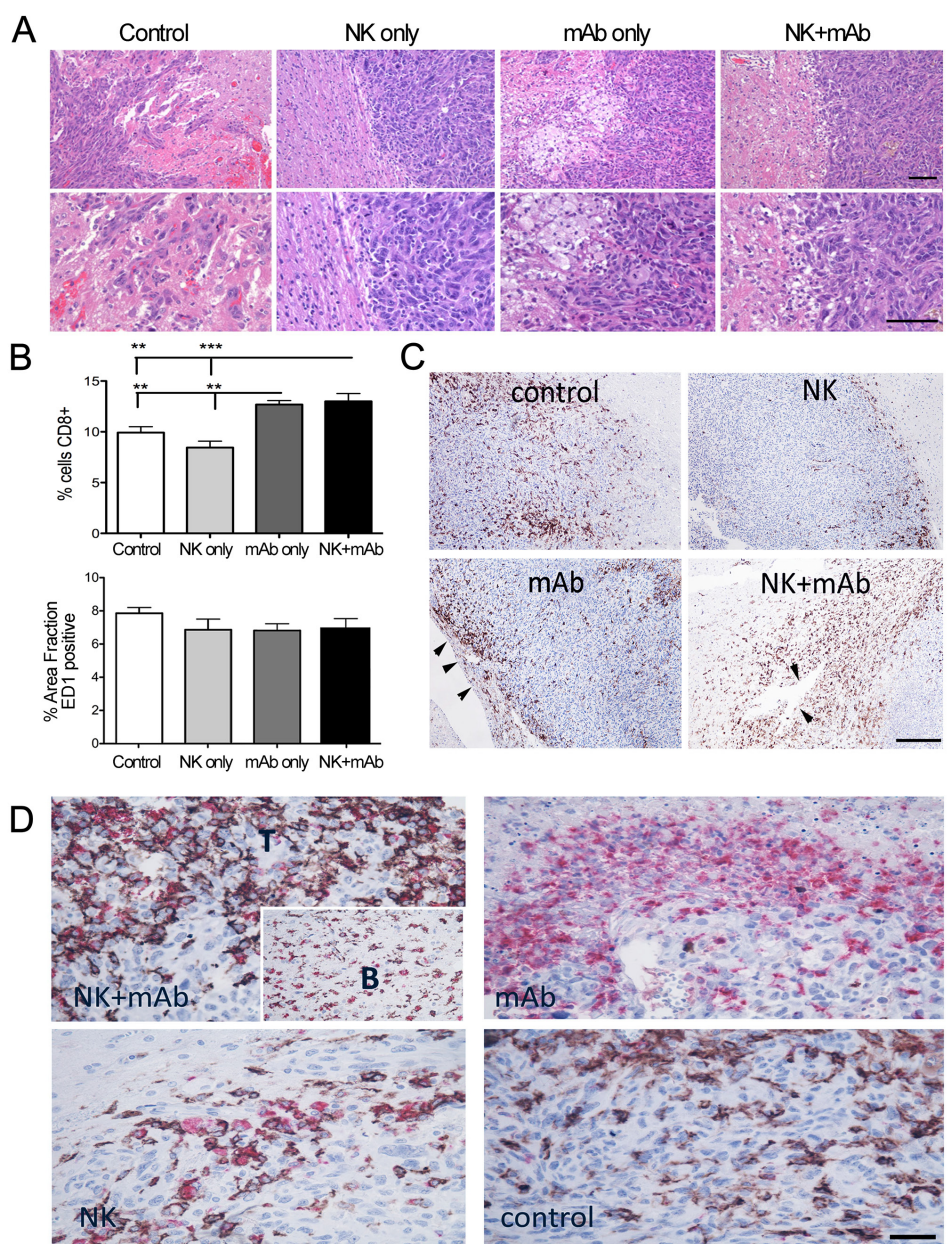
Differential macrophage/microglia phenotypes in response to treatment in U251-NG2 tumours (A) Upper panels, H&E staining showing tumor-brain boarders with varying amounts of large foamy cells (white) in response to mAb9.2.27 monotherapy and NK+mAb9.2.27 combination treatment (scale bar 200 μm, magnification 100 X). (A) Lower panels show higher magnification of the confrontation zone (scale bar 200 μm, magnification 200 X), (B) Upper panel show quantification of area fraction CD8^+^ cells in control, monotherapy NK cells, mAb9.2.27 as well as in NK+mAb9.2.27 combination treatment, lower panel shows quantification of area fraction ED1^+^ cells, in the same groups as above. Data represent mean ±SEM from all tumors in the groups. (C) Immunolabeling of CD8 shows abundant recruitment via vascular cuffs and choroids plexus in NK+mAb9.2.27 and mAb9.2.27 treatments, (arrowheads) respectively. Control and NK treated tumors show CD8^+^ cells that are restricted to tumor/brain periphery scale bar 200 μm, magnification 200X. (D) Double labeling for CD8 (brown) and ED1 (red) show abundant double positive macrophage/microglia in the tumor core (T) as well as those migrating from the brain (B, insert). mAb9.2.27 treated tumors show predominantly ED1 single positive, controls show CD8 single positive, and NK monotherapy shows fewer CD8/ED1 double positive cells that remain at the tumor/brain periphery (scale bar 100 μm, magnification 400 X).

Next, we aimed to identify the cell populations implicated and delineate the mechanisms mediating the therapeutic effect of the combination treatment. Histological analysis revealed striking clusters of large foamy cells at the periphery and within the core of the mAb9.2.27 and NK+mAb9.2.27 treated U251-NG2 tumors that were markedly diminished in the control and NK cell treated tumors (Fig. 2A). At perivascular cuffs and choroid plexus, CD8^+^ cells were most pronounced in the mAb9.2.27 monotherapy and NK+mAb9.2.27 treated tumors compared to the control (One-Way ANOVA F_11.0_, df=3, p=0.0011, n=5) and NK cell monotherapy tumors (One-Way ANOVA F11.0, p=0.0001, n=5), where the CD8^+^ cells were restricted to the tumor/brain periphery (Fig. 2B and 2C). No significant difference in the abundance of phagocytic ED1^+^ cells across the different treatment groups was found in the U251-NG2 tumors (Fig. 2B). However, the majority of CD8^+^ cells in the NK cell monotherapy and combination therapy tumors co-expressed ED1. Moreover, while CD8^+^ED1^+^ cells deeply infiltrated the combination treated tumors, these cells remained at the tumor/brain border in the NK cell monotherapy (Fig. 2D). In contrast, CD8^−^ ED1+ cells densely infiltrated the mAb9.2.27 treated tumors, while the control tumors contained predominantly CD8^+^ ED1^−^ cells (Fig. 2D). The tropism for CD8^+^ ED1^+^ cells in the combination treated tumors was also confirmed in the U87MG tumors (Supplementary Fig. 6A-D). CD8+ cells were more abundant in the NK+mAb9.2.27 treated tumors compared to monotherapy and controls (One-Way ANOVA _19.24_, p=0.0002, n=5, (Supplementary Fig 4C). Likewise, ED1^+^ cells were most abundant after NK+mAb9.2.27 treatment (Two-Way ANOVA t_3.928_, p=0.0003, n=5), (Supplementary Fig 4D). As CD8 and ED1 markers can be expressed by both microglia and macrophages [27] that are difficult to distinguish by immunohistochemistry, we systemically depleted macrophages by intraperitoneal injection of clodronate once a week for 4 weeks, starting at the day of the treatment.

### Macrophage depletion by clodronate treatment abolishes the therapeutic response in NK+mAb9.2.27 combination therapy

**Figure 3:**
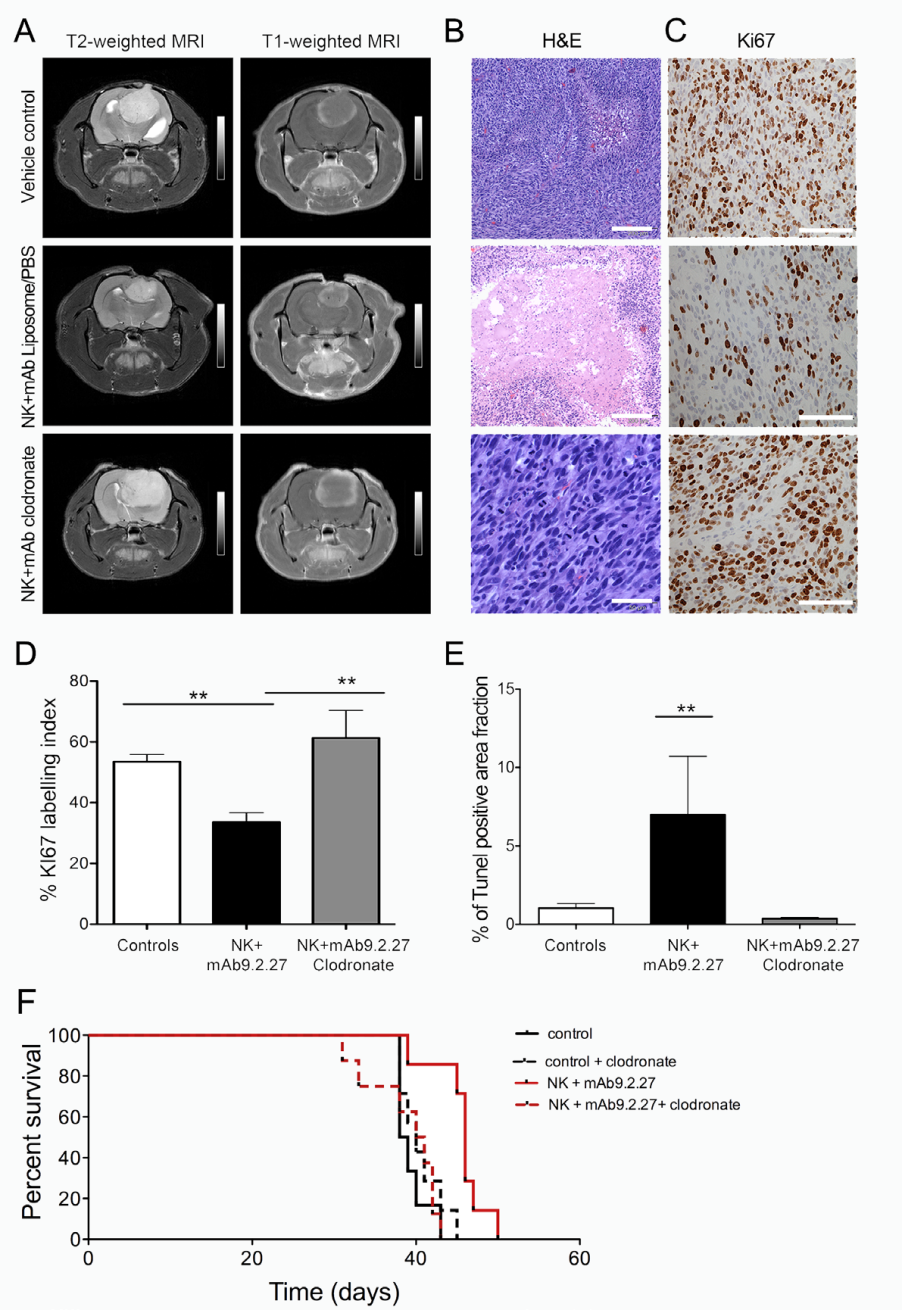
In vivo depletion of bone marrow derived macrophage abrogates therapeutic benefit from combined NK+mAb9.2.27 treatment (A) Longitudinal axial post-contrast T1-weighted and T2-Weigted MRI images of nude rats bearing P3-30 tumors treated with combination NK+mAb9.2.27, vehicle (PBS/liposomes) control, and NK+mAb9.2.27 treated animals given weekly intraperitoneal clodronate encapsulated liposomes. (B) H&E of representative animals from the vehicle control, NK+mAb9.2.27 combination and NK+mAb9.2.27+clodronate (Scale bar 200 μm, Magnification 100 X). (C) Representative Ki67 labeling in vehicle control, NK+mAb9.2.27 combination and NK+mAb9.2.27+clodronate treated animals (Scale bar 100 μm, Magnification 200X). (D) % Ki67 labeling index and (E) quantification of area fraction TUNEL positive apoptotic/necrotic cells. Data in (D) and (E) represent mean ±SEM of all tumors in the groups, **p<0.01 and *p<0.05. (F) Kaplan –Meier survival curves of NK+mAb9.2.27 treated and control tumor with or without macrophage depletion by clodronate, showing surviving fraction, n=8 animals/group.

To investigate further the mechanism leading to tumor regression, we utilized a unique GBM animal model developed in our laboratory where patient GBM biopsy spheroids are serially propagated *in vivo* over several generations. The resultant xenografts retain the genetic background, cellular heterogeneity and biological features of the original patient tumor [28]. P3-30 GBM cells express elevated levels of NG2/*CSPG4* (93.3±3.2 %, n=3), (Supplementary Fig. 1C). As observed with the U87MG tumors, the combination therapy diminished P3-30 lesions volumes including vasogenic edema on T2- and solid tumor sizes on T1-weighted MR imaging, respectively, compared to the vehicle treated control group. The macrophage depletion abrogated this therapeutic effect as indicated by increased lesion volumes including tumor and vasogenic oedema on T2-, as well as solid tumor volume on T1-weighted MR imaging respectively (Fig. 3A). The NK+mAb9.2.27 treated tumors had larger regions with tissue necrosis compared to the control and clodronate treated group that exhibited typical pseudopalisading necrosis and angiogenic vasculature (Fig. 3B). Moreover, the clodronate NK+mAb9.2.27 treated tumors had strikingly numerous mitotic figures and significantly increased tumor proliferation indicated by elevated Ki67 labeling index compared to NK+mAb9.2.27 treated animals (One-Way ANOVA F_11.19_, df=2, p=0.0011, n=5), (Fig. 3C-D). The macrophage depletion also significantly reduced the fraction of apoptotic/necrotic cells compared to vehicle treated controls, and NK+mAb9.2.27 tumors (One-Way ANOVA F _7.75_, p=0.0079, n=4), (Fig. 3B and 3E). The NK+mAb9.2.27 treatment prolonged animal survival compared to control, with median survival of 46 days *versus* 38.5 days respectively (Log Rank_10.06_, df=1, p=0.0015, n=7), (Fig. 3F). However, the macrophage depletion abrogated this increase of animal survival, with a median survival of 40.5 days (Log Rank 9.8, df=1, p=0.0017, n=8), (Fig. 3F). These results demonstrated the role of macrophages in therapeutic effect of NK+mAb9.2.27, as their depletion using clodronate diminished the animal survival.

### Macrophage depletion by clodronate treatment abolishes recruitment of ED1^+^ macrophage to the tumor core of NK+mAb9.2.27 combination therapy

**Figure 4:**
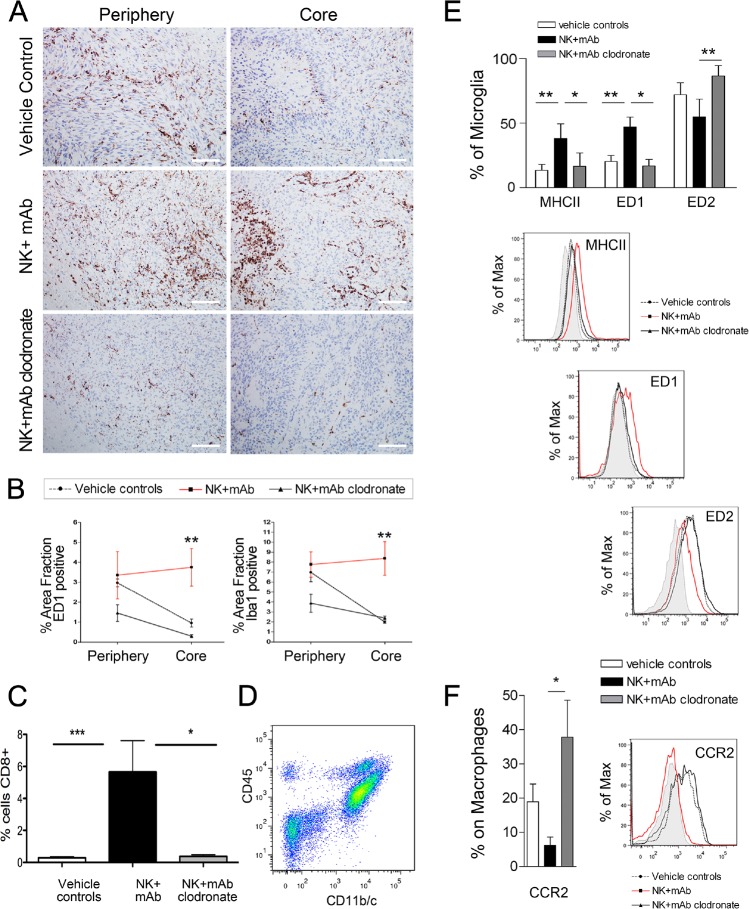
Increased recruitment of ED1+ activated macrophage/microglia in NK+mAb9.2.27 treated tumors (A) ED1 labeling in P3-30 bearing animals in the tumor core vs. periphery in vehicle control, NK+mAb9.2.27 combination and NK+mAb9.2.27+clodronate treated animals, (scale bar 100 μm, magnification 200 X). (B) The quantification of positive area fraction for ED1 and Iba1 in tumor core and periphery in vehicle control, NK+mAb9.2.27 combination and NK+mAb9.2.27+clodronate treated animals. (C) Quantification of CD8^+^ area fraction in vehicle control, NK+mAb9.2.27 combination and NK+mAb9.2.27+clodronate treated animals (D) Flow cytometric representative dot plot showing the gating of cell populations from rat brain cell suspension expressing CD11b/c and CD45, permitting the differentiation of macrophages (CD45^high^CD11b/c^+^) and microglia (CD45^low^CD11b/c^+^). (E) Upper panel, % microglia cells expressing MHC class II, ED1 and ED2. Lower panels, mean fluorescence intensity histograms as % of Max of data in (E). (F) Percentages of CCR2^+^ macrophages (right) and mean fluorescence intensity as % of max for CCR2 (left). The data in (B), (C), (E) and (F) represent mean ±SEM from all tumors in the groups, ***p<0.001, **p<0.01 and *p<0.05.

The NK+mAb9.2.27 treatment increased the tropism of ED1^+^ cells into the tumor core compared to vehicle treated controls (Two-Way ANOVA t_3.991_, p=0.013, n=4) and macrophage depleted NK+mAb9.2.27 treated tumors (Two-Way ANOVA t_4.276_, p=0.01, n=4), (Fig. 4A and 4B). Iba1, the calcium binding protein expressed by activated and phagocytic macrophage/microglia was also elevated in the core of the combination treated tumors compared to vehicle treated control (Two-Way ANOVA t_4.633_, p=0.01, n=4), and macrophage depleted tumors (Two-Way ANOVA t_3.235_, p=0.0243, n=4), (Fig. 4B). Significant numbers of CD8^+^ cells were recruited into the tumor microenvironment after the NK+mAb9.2.27 treatment (One-Way ANOVA t_7.482_, p=0.0001, n=5), (Fig. 4C), as was the case for U87MG and U251-NG2 tumors. Remarkably, macrophage depletion by clodronate injection abrogated this ED1 recruitment compared to NK+mAb9.2.27 treated animals (Two-Way ANOVA t_34.276_, p=0.013, n=4). The CD8^+^ cells recruited to the NK+mAb9.2.27 treated tumor were significantly elevated compared to controls and NK+mAb9.2.27 with macrophage depletion (One-Way ANOVA _18.5_, p=0.0001, n=5), (Fig. 4A-C).

### NK+mAb9.2.27 combination therapy promotes the recruitment of macrophage and microglia with a pro-inflammatory phenotype

In order to investigate the subtype of cells expressing ED1 and CD8, we further investigated the phenotype of brain immune cells by 9 colors multiparametric flow cytometry on brain cell suspensions obtained from P3-30 controls, or NK+mAb9.2.27 treated tumors with or without clodronate administration. The cellular populations representing macrophages (CD45^high^CD11b/c^+^) and microglia (CD45^low^CD11b/c^+^) were distinguished (Fig. 4D). We could demonstrate that microglia increased ED1 expression after NK+mAb9.2.27 treatment, compared to vehicle treated controls and concomitant NK+mAb9.2.27 and clodronate treatment (Two-way ANOVA t_3.64_, p<0.001, n=6 and t_3.33_, p<0.05, n=4), (Fig. 4E). In the other hand, the NK+mAb9.2.27 combination treatment significantly increased MHC class II expression by microglia compared to controls (Two-way ANOVA t_3.7_, p<0.01, n=6), (Fig. 4E). In addition, while microglia from NK+mAb9.2.27 treated tumors expressed low levels of the scavenger receptor ED2, microglia from macrophage-depleted and untreated animals had significantly increased ED2 levels (Two-way ANOVA t_3.45_, p<0.01, n=6). We observed that 60 % of brain macrophage expressed ED1 following NK+mAb9.2.27 combination treatment, which correspond to its expression in control animals. The proportion of macrophages expressing CCR2 was significantly decreased after NK+mAb9.2.27 therapy (Two tailed T-test, p=0.042, n=6), (Fig. 4F). However, both microglia and macrophage did not express CD8 extra-cellularly. These data might indicate that the therapeutic efficacy of NK+mAb9.2.27 combination treatment was mediated by increased recruitment of ED1^+^ CCR2^low^ macrophages, which then differentiate into (or activate) ED1^+^ ED2^low^MHCII^high^ microglia in the tumor microenvironment. These phenotypic modifications of macrophage/microglia suggested the elaboration of a pro-inflammatory environment following the NK+mAb9.2.27 treatment. As evidence we found that the classical T-helper 1 pro-inflammatory cytokines, IFN-γ and tumor necrosis factor alpha (TNF-α) were up-regulated in the cerebrospinal fluid (CSF) from the NK+mAb9.2.27 combination-treated animals whereas the immunosuppressive cytokine IL-10, as well as IL-6 and IL-1β were more abundant in the CSF from the untreated animals (Table 1). In addition, although not statistically significant, in the plasma there was a tendency for decreased IL-10 (38±25.5pg/ml, n=7 vs. 2.4±2.4pg/ml, n= 6) and TNF-α (25.6±14.2pg/ml, n=7 vs. 0pg/ml, n= 6) levels following treatment.

**Table 1:** Concentration of cytokines in CSF of vehicle control and NK+mAb9.2.27 treated rats Concentrations of IL-1α, IL-6, IL-10, IFN-γ and TNF-α are given in pg/ml.

	Vehicle Control			NK+ mAb9.2.27		
	Mean	SEM	n	Mean	SEM	n
IL1-α	684.5	290.2	2	50.2	29.0	3
IL6	4593.0	2256.7	2	125.2	125.2	3
IL-10	25617.9	25617.9	2	0.0	0.0	3
IFN-γ	0.0	0.0	2	21.9	13.5	3
TNF	0.0	0.0	2	110.9	100.9	3

### mAb9.2.27 and IFN-γ-activated microglia are more cytotoxic to GBM cells than NK cells

**Figure 5:**
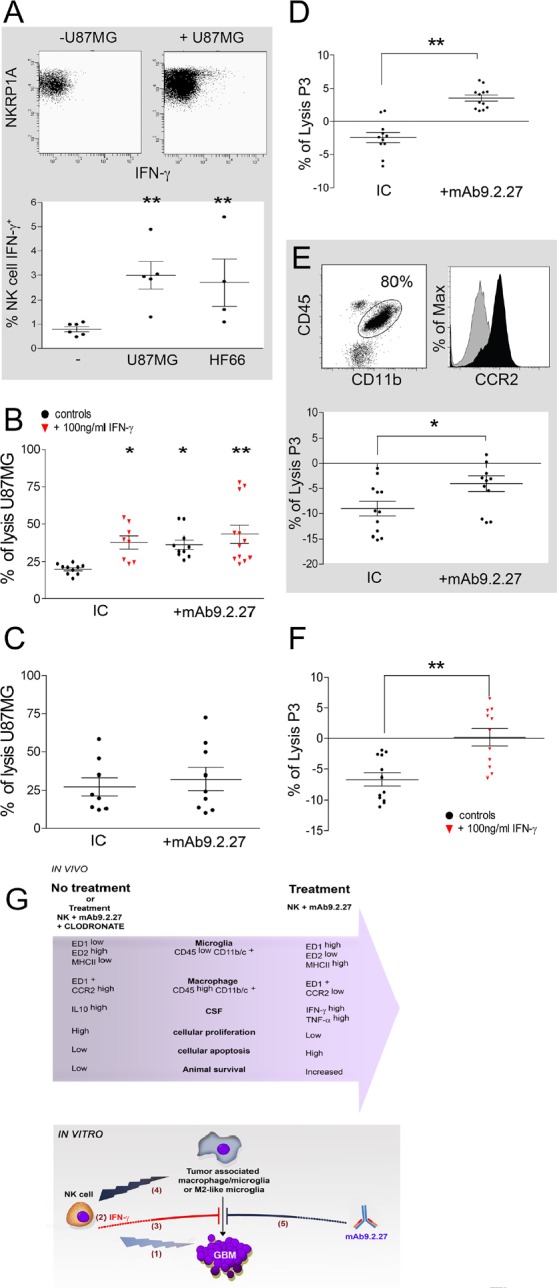
IFN-γ and mAb9.2.27 increase cytotoxicity of microglia against GBM (A) Upper panel: dot plot showing IFN-γ secretion in IL-2 activated NK cells in contact with U87MG cells, Lower panel: quantification of IFN-γ secretion in IL-2 activated NK cells co-cultured with U87MG or HF66 at ratio 2/1 for 18 h. (B) % lysis of U87MG *in vitro* by microglia from naïve LEWIS rats following activation with IgG2a isotype control, IFN-γ or/and mAb9.2.27. (C) % lysis of U87MG *in vitro* by NK cells in the presence of IgG2a isotype control or mAb9.2.27. (D-E) *Ex vivo* purified rat (D) and patient (E) macrophage/microglia from tumor microenvironment were investigated for cytotoxicity against P3-30 tumor pre-incubated or not with mAb9.2.27. Human TAMs were phenotyped for CD45^+^CD11b^+^CCR2 expression (E dot plot: CD45+ against CD11b+. E Histogram: isotype control grey histogram, CCR2 black histogram). Lower panel: % lysis of P3-30 tumor by *ex vivo* TAM from patient GBM following activation with IgG2a isotype control, or mAb9.2.27. (F) Cytotoxicity of rat TAMs was also investigated after 96 h (F) incubation with 100 ng/ml of IFN-γ. Cell viability was determined by flow cytometry using TOPRO-3 as supravital dye. Data are plotted as mean ±SEM, **p<0.01, *p<0.05. (G) Adoptively transferred NK cells as initiators of GBM destruction: schematic representation of the in vivo and in vitro experimental findings. ***In vivo*: combination NK+mAb9.2.27 treatment leads to increased infiltration of the tumor by microglia and macrophages with pro-inflammatory phenotypes, with respectively ED1^high^ ED2^low^ MHC class II^high^ and ED1^+^ CCR2^low^ molecular expression**. This was associated with increase of pro-inflammatory cytokines in the CSF of NK+mAb9.2.27 treated animals, as well as diminution of cellular proliferation and increased tumor cell apoptosis. This resulted in increased animal survival that was abolished by depletion of systemic macrophages by injection of liposome-encapsulated clodronate. ***In vitro*: activated NK cells induced cellular cytotoxicity against GBM (1)**. The NK cells/GBM interaction led to IFN-γ secretion by NK cells (2). This cytokine inhibited tumor survival promoted by tumor-associated macrophages/microglia (3). NK cells efficiently killed anti-inflammatory M2-like microglia (4). Moreover, mAb9.2.27 inhibited tumor survival mediated by tumor associated macrophages/microglia (5).

In order to investigate the mechanisms of the synergistic mAb9.2.27 and NK cell cross-talk with microglia, the cytotoxicity of resting microglia and NK cells with and without mAb9.2.27 was tested in NG2/*CSPG4* positive GBM cells *in vitro*. First, we observed that activated NK cells in contact with GBM cells produced IFN-γ, as revealed by intracellular flow cytometry (Two tailed T-test, NK cells alone *vs.* NK cells+U87MG p=0.0043, n=5 and *vs.* HF66 p=0.0095, n=4), (Fig. 5A). This was corroborated by increased IFN-γ and TNF-α released into the supernatant culture medium from NK cells in contact with GBM cells compared to supernatants from tumor cells only, (IFN-γ: One-Way ANOVA_6.54_, p=0.03; n=11; and TNFα: One-Way ANOVA _9.59_, p=0.0083; n=11). Correspondingly, the killing capacity of resting microglia against U87MG GBM cells was increased by overnight activation with IFN-γ and against target pre-incubated with mAb9.2.27 compared to the cytotoxicity of resting microglia with IgG2a isotype control (IC) (One-Way ANOVA, p<0.05, n=5 and p<0.05, n=4 respectively), (Fig. 5B). Interestingly, there was no cumulative effect of IFN-γ and the mAb9.2.27 on the cytotoxicity of microglia. Indeed the cytotoxic capacity of microglia following activation with IFN-γ in absence of mAb9.2.27 was not significantly different from their killing capacity in presence of this mAb, with respectively 37.8 ±4.2 % and 43.2±5.9 % U87MG lysis (Fig. 5B). In contrast, there was no significant difference between cytotoxicity of IL-2 activated NK cells in the presence or absence of mAb9.2.27 against all GBM cell lines tested (Fig. 5C). The cytotoxicity of microglia and activated NK cells was also tested against the HF66 GBM cells lines, recognized by mAb9.2.27 at almost 100 %, and the previous results with U87MG were confirmed. Activation of microglia by IFN-γ, overnight or during 5 days rendered them highly cytotoxic against GBM, whereas culture with GBM conditioned medium promoted the survival of HF66 GBM cells, consistent with microglial M1- and M2-like physiology and phenotype, (Supplementary Fig. 5A and B respectively). As was the case for U87MG, the mAb9.2.27 abrogated the survival of HF66 tumor cells, mediated by M2-like differentiated microglia (Supplemental Fig. 5B). Moreover, NK cells preferentially killed the differentiated M2-like microglia, whereas M1-like microglia was more resistant to NK cell lysis (Supplementary Fig. 5C). Collectively, these data indicate that mAb9.2.27 could mediate ADCC by microglia but not by NK cells *in vitro*.

### mAb9.2.27 and IFN-γ abrogate tumor-promoting effect of tumor-associated macrophage/microglia *ex-vivo*

Since our *in vivo* results demonstrated that mAb9.2.27 monotherapy slowed down tumor growth in the initial phases, we investigated whether mAb9.2.27 could impact on macrophage/microglia function directly. These macrophage/microglia were purified from the brain of P3-30 tumor bearing rats and examined for their cytotoxic capacity *ex vivo* against P3-30 cells pre-incubated or not with mAb9.2.27, and after 18 h or 96 h of culture in the presence of IFN-γ. The TAM promoted P3-30 tumor survival *ex vivo*, indicated by a diminution of its spontaneous death in culture, presented as a negative percentage of lysis (Fig. 5D). P3-30 tumor cells became susceptible to rat TAMs cytotoxicity in the presence of mAb9.2.27 (One-Way ANOVA, p<0.01, n=6), (Fig. 5D). Remarkably, this effect was reproduced using patient GBM derived TAM (Fig. 5E) defined as CCR2^high^ (Fig. 5E histogram) indicating the robust capability of mAb9.2.27 to inhibit tumor promoting effect of TAMs. Finally, although rat TAM failed to inhibit the tumor survival after 18 h in culture with IFN-γ, a prolonged activation to 96 h did significantly inhibit tumor survival (One-Way ANOVA, p<0.01, n=4), (Fig. 5 F). The conclusions from the *in vivo* and *in vitro* studies are summarized in the schematic figure 5G.

## DISCUSSION

It is established that gliomas, like many cancers, develop multiple strategies to evade detection and destruction by the immune system. One of these mechanisms involves the recruitment of macrophages/ microglia and their maintenance in an anti-inflammatory (M2-like) state, through the secretion of tumor-associated factors likeTGF-β or prostaglandin E_2_, which promote tumor growth, migration and angiogenesis [29]. Many new therapeutic strategies have the ambition to skew M2-like TAM to M1-like phenotypes with anti-tumoral properties [30, 31]. We investigated a novel combination immunotherapeutic strategy to treat GBM using intratumoral administration of mAb9.2.27 against human NG2/*CSPG4* with activated NK cells.

We treated human U87MG and U251-NG2 GBM cell lines xenografted in athymic nude rats. While the treatment significantly increased survival in both models, the U251-NG2 tumors exhibited only partial responses. As mAb9.2.27 labeled almost 3-fold less U251-NG2 cells than U87MG cells, the modest effect of the combination treatment on the U251-NG2 tumors might be expected and reinforces the specificity of the mAb9.2.27 in the therapeutic response. Moreover it emphasizes the importance of the presence of an interaction between mAb9.2.27 and NG2/*CSPG4* antigen in future clinical applications. In addition, we used a more relevant model based on rats bearing patient derived GBM biopsy established to be more therapy resistant due to their high cellular heterogeneity. Although several mouse models of brain tumors have been engineered by altering signaling pathways that are disrupted in human gliomas (reviewed in [32]), our patient derived xenografts retain the cellular heterogeneity, invasive, angiogenic and comparative genomic hybridisation (cGH) gene profiles as the original tumor [33]. Our results demonstrated that NK+mAb9.2.27 treatment led to increased animal survival through the recruitment of ED1 positive cells via the choroid plexus into the tumor, in addition to increased numbers of cells with heightened capacity to secrete cytotoxic components such as myeloperoxidase, granzyme B and IFN-γ. The difference in efficacy between the U87 and P3-30 combination treated xenografts might be expected when treating homogenous cell line *versus* heterogenous, patient-derived GBM xenografts. However, confirming therapeutic efficacy using different model systems adds robustness to our findings. The combination treatment prolonged survival of P3-30 xenografts from 39 to 46 days, representing an increase in survival of 18 %. However, the median survival after GBM treatment in contemporary patient studies is 14.6 months. Thus, this 18 % increase amounts to 2.5 months if extrapolated to a clinical setting. When we consider the very few and small improvements in prognosis for GBM patients over the last 5 decades, this 18 % longer survival might indeed have clinical significance and realistically highlight the challenges of treating human cancers with high degree of heterogeneity.

Furthermore, we could demonstrate that the combination treated animals had elevated concentrations of the pro-inflammatory cytokines IFN-γ and TNF-α in their CSF and diminished levels of anti-inflammatory cytokines such as IL-10 compared with control animals. These results indicated that the intra-tumoral treatment of NK+mAb9.2.27 modified the tumor microenvironment from anti-inflammatory to pro-inflammatory one resulting in tumor regression. This massive recruitment of inflammatory cells into the tumor is reflected by the MR images at 3 and 4 weeks in the U87MG tumors *in vivo*, where increased lesion size could be demonstrated by T1-weighted MRI. Smith et al. [34] previously reported this phenomenon as the flare effect following intralesional immunotherapy with activated lymphocytes and IL-2, in patients with recurrent high grade astrocytomas [34]. Apparent worsening MRI imaging directly after therapy indicated by increased nodular enhancement, edema, and mass effect [7, 34] characterized the flare effect. This phenomena was also reported following gene therapy [35], convection enhanced delivery of cytokines [36] or after placement of the GliaSite radiation therapy system [37] to treat brain tumors. The flare effect highlights the challenges of using the Macdonald's radiological response criteria for assessing therapeutic efficacy after immunotherapy [38]. The immune cell recruitment characterized by hyper-intense lesions on T1-weighted MRI might easily be misinterpreted as tumor progression whereas in reality, it represents a pseudo-progression. Other measures of immune responses have been demonstrated as independent predictive factors for favorable immune responses after immunotherapy [39]. Paradoxically, vitiligo is still reported as an adverse event in clinical trials evaluations, however, its presence strongly portends enhanced survival in melanoma patients. More research is required to develop clinically reliable methods for evaluating response to successful immunotherapy.

In contrast to untreated animals, microglia from the NK+mAb9.2.27 combination treated animals up-regulated MHC class II molecules and ED1 expression, rendering them competent for presentation of GBM antigens [40]. In addition, microglia expressing ED2 scavenger receptor were diminished by the combination NK+mAb9.2.27 treatment, indicating a reduction of perivascular microglia postulated to facilitate GBM progression [41]. In parallel the NK+mAb9.2.27 treatment decreased the proportion of macrophages expressing CCR2, a subpopulation already proven to promote GBM invasiveness [42]. These characteristic features of activated cytotoxic cells, as well as the increased survival were abrogated by depletion of circulating macrophages with clodronate treatment. This indicated that the beneficial outcomes were mediated by the recruitment of pro-inflammatory macrophages from the periphery to the tumor. Nevertheless, we cannot exclude the possibility that dendritic cells play a role in the therapeutic efficacy, as liposome-encapsulated clodronate was shown to selectively induce apoptosis of macrophages [43] and phagocytic dendritic cells [44]. Indeed, both cell types share the expression of CD45^high^CD11b/c^+^ and only OX-62 can distinguish dendritic cells from macrophages [45-47]. Unfortunately, we did not use anti-OX-62 (CD103) mAb in our flow cytometry staining to be able to differentiate MHC class II positive activated macrophages from dendritic cells. Taken together our results indicate that the combination treatment permits the infiltration of ED1^+^ CCR2^low^ macrophages, which then differentiate into or promote ED1^+^ ED2^low^MHC II^+^ smicroglia that favor tumor destruction by creating pro-inflammatory tumor microenvironment.

We propose that implantation of activated NK cells was the decisive element required for the development of the pro-inflammatory environment. Neither NK cells nor mAb9.2.27 monotherapy significantly prolonged survival, but since combination treatment did, we suggest that a synergistic action of NK cells and mAb9.2.27 facilitated tumor regression. Recently, NK cells were demonstrated to mediate the therapeutic efficacy by ADCC of a chimeric antibody directed against an intracellular antigen, phosphatase of regenerating liver-3 (PRL-3), in nude mice. Depletion of NK cells with asialo GM-1 antibodies abolished the therapeutic efficacy [48] underscoring the therapeutic impact of NK cell induced ADCC. Using T1-weighted sequences we established in the present study that the mAb9.2.27 treated tumors exhibited reduced leakage of contrast agent from the tumor vasculature. This effect of mAb9.2.27 is likely due to blocking the angiogenic potential of NG2/*CSPG4* expressing pericytes on the tumor vasculature. Although some provocative studies reported that immature GBM stem-like cells are capable of differentiating into functional vascular endothelium and might contribute to tumor angiogenesis[49, 50]recent systematic studies demonstrated using chromogenic *in situ* hybridization on intact tissue that neoplastic cells are a rare component in human GBM microvasculature, do not incorporate into the vessel wall or express CD34 [51], Thus, blocking NG2/ *CSPG4* signaling may have decreased the angiogenic capacity of the tumors mediated by pericytes. We have previously shown that NG2/*CSPG4* was located on the tumor cells as well as their angiogenic vasculature on pericytes [8, 52]. Others have demonstrated that targeting NG2/*CSPG4* on these pericytes diminished mouse pathological retinal and corneal angiogenesis [53]. Recently, Wang et al. demonstrated in a xenograft model of breast cancer that the administration of the anti-NG2/*CSPG4* mAb225.28 diminished tumor growth, associated with reduced proliferation and vascular density, and increased apoptosis [13]. Moreover, we proved that purified GBM associated macrophage/microglia, from rat and acutely dissociated patient biopsy, were no longer able to promote tumor survival in presence of mAb9.2.27 *ex vivo*. It would be interesting to study further this capacity of mAb9.2.27 to modulate macrophage polarization and to examine the mechanisms involved. Numerous studies have reported that ligation of the Fc receptor induces the transcription of several genes in macrophages that contribute to subsequent inflammatory and immune responses [54]. The mechanisms of macrophage/microglia ADCC against brain tumors are not well documented, nonetheless a study from the 90's demonstrated microglia ADCC using an anti-EGFR mAb [55]. Furthermore, using an FcγR deficient mouse model of brain metastatic B16 tumor expressing EGFRvIII, Sampson et al. demonstrated that the survival advantage of anti-EGFRvIII mAb treated animals was FcγR dependent [56]. As resident macrophage/microglia are known to express FcγR [57], these findings support our data implicating a subpopulation of macrophage/microglia in the tumor destruction. Since NK cells alone were not able to induce potent ADCC, but were required for the synergistic effects of the combination therapy, we hypothesized that NK cells could be involved in generation of tumor immunoediting, as recently described by O'Sullivan et al. [23]. We demonstrated that activated NK cells were able to secrete TNF-α and IFN-γ in contact with GBM. However, the direct effect of TNF-α expressing GBM cells is unclear since we previously demonstrated that these cells were resistant to TNF-α mediated apoptosis due to augmented PI3K/Akt and NFκB survival signaling [13]. Indeed, the Fernandez-Luna group showed that blockade of NFκB in glioma initiating cells (GICs) with genetic strategies or small molecule inhibitors induced cell cycle arrest, cellular differentiation and senescence [58]. However as these effects were described for these *so-called* GICs, the implications of their findings remain to be proven since the ontogeny of GBM from GICs and the extent to which GBM cells are capable of undergoing senescence remain controversial. Interferon-γ was previously shown to switch human immunosuppressive TAMs into immunostimulatory cells [59], to upregulate their expression of MHC class II [60] and co-stimulatory molecules [61]. The macrophages were subsequently more phagocytic [62] and released more cytotoxic pro-inflammatory cytokines [59, 63], nitric oxide [28] and MPO [64]. In this study we found that IFN-γ may also inhibit the survival promoting effect of TAMs on P3-30 GBM *in vitro*. However it is difficult to translate this finding *in vivo* as we may have used a high concentration of IFN-γ *in vitro* (100 ng/ml) compared to the concentration we found in the CSF (20 pg/ml). Indeed, lower levels of IFN-γ (1 to 20 ng/ml) were shown to promote microglia neurogenesis while high concentrations could be neurotoxic (50 ng/ml) [65, 66]. Future studies should address this question. In addition, we showed that NK cells preferentially killed the differentiated M2-like microglia, whereas M1-like microglia were more resistant to NK cell lysis. These results are similar to those from a recent work of Bellora et al. showing that mouse activated NK cells preferentially kill M2 macrophages rather than M1 macrophages [67]. To summarize, our findings demonstrate that activated NK cells could modulated the anti-inflammatory tumor microenvironment to a pro-inflammatory contexture facilitating the recruitment and the differentiation of cytotoxic and inflammatory macrophage/microglia first by secretion of pro-inflammatory cytokines and then by the destruction of anti-inflammatory tumor associated macrophage/microglia. As we utilized athymic nude rats, the possible influence of regulatory T cells could be underestimated. Nonetheless, to develop effective immunotherapy strategies for aggressive GBM tumors necessitates use of preclinical models that exhibit similar cellular heterogeneity, invasive behavior, and express appropriate tumor antigens. Our patient derived P3-30 GBM model fulfills these requirements, however requires propagation in athymic rats. Apart from diminished T cells, the other immune cells are representative and fully functional [68]. The alternative to xenotransplantation would have been to use syngeneic models, where the entire immune system is intact. However, as we are targeting human NG2/*CSPG4* with an anti-human mAb9.2.27, this model would not be appropriate due to poor recognition of NG2/*CSPG4* from rat origin by mAb9.2.27. Since pro-inflammatory microglia are implicated in several brain diseases [69, 70], future work will have to verify the safety and tolerability of our combination treatment prior to clinical application. In a potential future clinical trial, we envision that our combination NK+mAb9.2.27 immunotherapy would be administered locally after surgical debulking and concurrent temozolomide and radiotherapy treatment. In conclusion, administering NK cells as adjuvant treatment might be a useful strategy for increasing the therapeutic efficacy of several mAb-based passive immunotherapies and thereby positively impact the survival of GBM patient.

## MATERIALS AND METHODS

### Tumor cell lines and their culture, Patient Biopsy

The human GBM cell line U251-NG2 over-expressing NG2/*CSPG4* [8] was originally derived from U251. The U251, U87MG (American Type Culture Collection, Rockville, MA) and HF66 (Ford Cancer Centre, Detroit, MI) GBM cell lines were propagated as previously described [8]. The GBM biopsies were obtained from surgical resections performed at Haukeland University Hospitals. The local ethical board (REK Vest) and the Data Protection Agency in Norway approved the collection of tumor tissue. Patients gave their informed consent to specimen collection for research purposes and their samples were analyzed anonymously.

### Antibody PEGylation

The azide free mAb9.2.27 (10mg/ml) against the cell surface chondroitin sulphate proteoglycan NG2 was a generous gift from Professor Reisfeld (Scripps Research Institute, La Jolla, San Diego, CA). Since the mAb9.2.27 is an anti-human mouse IgG2a isotype known to bind weakly to Fc receptors on effector cells [71], polyethylene glycol (PEG) was conjugated to the mAb in order to increase its interaction with the Fc receptor (FcγRIII) on the effector cells [72]. An aliquot of this antibody was PEGylated by 10 times dilution with a 24 % PEG 20,000 (Sigma Aldrich, Bornem, Belgium) solution (DMEM supplemented with 10 % FBS) for at least 30 min at 4°C. The pegylated reaction was mixed with a 4 X Tris-HCl 0.1 M Buffer (pH 7) containing 4 % SDS, 40 % Glycerol, 0.001 % Bromophenol Blue and 1 % Beta-Mercaptoethanol and then charged in a 7.5 % acrylamide gel and electrophoresis was performed at 60 mV/gel in SDS-PAGE system. We demonstrated that the mAb9.2.27 was successfully PEGylated by approximately 2 molecules of the 20 kDa PEG, as revealed by Comassie blue staining (Supplementary Fig. 6A). To investigate the ability of mAb9.2.27 to crosslink the NK cell FcγRIII, NK cells were pre-armed with PEGylated mAb9.2.27, native mAb9.2.27 or alone and analyzed by flow cytometry. Only pre-arming NK cells with PEGylated mAb9.2.27 facilitated ligation of the FcγRIII. Incubation with native mAb9.2.27 or 24 % PEG alone did not result in specific recognition of NK cell FcγRIII (Supplemental Fig. 6B). We also investigated the ability of the mAb9.2.27 to crosslink the FcγR on purified microglia, the major brain effectors. The PEGylated mAb9.2.27 had greater affinity for microglia compared to NK cells, and other controls (Supplemental Fig. 6C).

### Animals and intracranial implantation

8-10 week old immunodeficient athymic nude rats were bred from Hsd:RH-Foxn1 rnu/+ female and Hsd:RH-Foxn1 rnu male breeding pairs. Rats of both sexes were used for tumor implantation and were maintained as previously described [8]. This athymic nude rat strain has autosomal recessive mutation on rnu locus of chromosome 10 [73]. It is deficient for some T-cell subpopulations, but has normal B-cell function [74], increased NK cell number [75] and macrophage cell populations [74]. 7-11 week old immunocompetent LEWIS (LEW/Han^™^ Hsd; Harlan, Horst, The Netherlands) male rats were also used for *in vitro* assays. All animal procedures were performed in accordance with protocols approved by The National Animal Research Authority (Oslo, Norway). A burr-hole was made 1 mm posterior to the bregma and 3 mm to the right of the sagittal suture using a micro-drill with a bit diameter of 2.9 mm. A Hamilton syringe with inner diameter of 810 μm was introduced to a depth of 2.5 mm below the brain surface, and the spheroids were slowly injected and the syringe left in place for 3 min before withdrawal.15 tumor spheroids (each containing 30,000 cells) were selected under a stereo light microscope. 25 animals were xenografted with U251-NG2, 28 animals with U87MG and 28 animals with patient GBM biopsy spheroids from patient 3 (P3-30) that had been serially *in vivo* passaged as previously described [76]. The skin was closed with an Ethilon 3-0 suture. The tumors were allowed to grow for 3 weeks prior to intra-lesional treatment with mAb9.2.27, NK cells from littermates (2 million cells) or combined NK+mAb9.2.27. Antibodies and NK cells were administered using a 26-gauge cannula connected to an infusion catheter of an osmotic mini pump (Alzet Inc.,) or a syringe, respectively, to the same coordinates as were used for the tumor implantation procedure. The animals were sacrificed at the onset of neurological symptoms of lethargy and paralysis in order to obtain overall survival information. An additional cohort of 31 animals xenografted with P3-30 spheroids and treated with NK+mAb9.2.27 with or without liposome-encapsulated clodronate was sacrificed at set time points as controls by CO_2_ inhalation and decapitation. Their brains were removed, dissociated into single cell suspensions or fixed in 4 % formalin for the characterization of immune cells infiltrating the brain by flow cytometry and/or immunohistochemistry.

### Macrophage depletion by clodronate treatment

Depletion of macrophages was done by intraperitoneal injection (IP) of 1 ml/100 g body weight using 5 mg/ml of liposome-encapsulated clodronate (Encapsula NanoScience). The injections were repeated weekly for 4 weeks after treatment or until development of neurological symptoms. Control rats were injected with same volume of PBS or control liposome at the same time. Depletion of macrophages was confirmed on splenocytes for P3-30 xenografted rats by flow cytometric determination of ED1 and ED2 positive cells (supplementary Fig. 7A and 7B). It should be noted that the IP injection of liposome-encapsulated clodronate could not deplete resident microglia (Supplementary Fig. 7C). It was demonstrated by several groups that the microglia depletion could only be achieved by intracranial administration of liposome-encapsulated clodronate [77, 78]. Indeed, we drastically decreased the CD11b/c^+^CD45^high^ macrophages recruited to the brain in context of brain tumor, without major modification of CD11b/c^+^CD45^low^ microglia population (Supplementary Fig 7C).

### Convection enhanced delivery of mAb9.2.27

Azide free mAb9.2.27 (4 mg/ml in 24 % PEG solution) and IgG2a isotype controls were administered through a 26-gauge cannula connected to an osmotic mini pump (AD01; Alzet Inc., Mountainview, CA). The pumps were installed into the same coordinates as the tumor using a stereotactic frame and infused at a CED rate of 8 μl/h over 24 h.

### Magnetic resonance imaging

MRI images were obtained with a 7 Tesla Bruker Pharmascan (Bruker Biospin, Ettlingen, Germany) using a 38 mm rat head volume coil for transmitting and receiving as previously described [79]. T1-weighted MR imaging was obtained using a RARE (rapid acquisition relaxation enhancement) sequence with TR = 1.300 ms, TE = 8.86 ms, rare factor = 4, matrix size = 256 × 256, pixel size = 137 × 137 μm^2^, a slice thickness of 1.5 mm (eight slices, no gap) and 6 averages. 0.2 mmol/kg Omniscan® contrast agent (gadodiamide, MW 0.58 kDa, GE Healthcare, Norway) was injected through the tail vein.

### Histology, immunohistochemistry and quantification

Brains were formalin fixed, paraffin embedded, and every 20^th^, 3-5μm thick tissue section was stained with haematoxylin and eosin. Consecutive sections were immunohistochemically stained with various mAb: anti-IFNγ (AbDSerotec, Oxford, UK) anti-MIB-1 (Ki67, Dako, Glostrup, Denmark), rat specific anti-CD8 (OX-8, AbDSerotec,), anti-CD68 (ED1, AbDSerotec), anti-granzyme B, polyclonal anti-myeloperoxidase, anti-CD3 (Dako), using the ABC method with 3, 3”-Diaminobenzidine (DAB) and H_2_O_2_(DCS, Hamburg, Germany). Percentages of CD8, Granzyme B, and MPO+ immune cells against the negative, haematoxylin counterstained nuclei were quantified in at least 10 fields of view/per section for all the animals in each group. Immunohistochemically stained sections were segmented and quantified with the use of CellProfiler software package [80] and custom made image analysis pipelines. Hematoxylin and DAB staining were separated by the use of the UnmixColors module. Thereafter the CorrectIlluimination module applied an illumination correction in order to adjust uneven light illumination of the image. Hematoxyllin stained nuclei and positive DAB stained cells were identified using the IdentifyPrimaryObjects module with the Otsu Global threshold method. For assessing the precision of the segmentation, the images were saved with an overlay of the segmentation masks. For further details, the specific custom-made pipelines used with CellProfiler are attached in the supplementary files.

ED1 positivity was assessed using morphometry with NIS Elements v 4.0 software and Nikon Eclipse 600 microscope (Nikon). Semi-quantification of DAB staining by morphometry on immunohistochemistry was based on signal intensity of ED1 positivity relative to negative control section that was set as threshold. The threshold was stored and subsequently applied with identical microscope settings for scoring of all tumor sections. Immune positive elements (pixels above threshold) were measured and expressed as area fraction of the visual field for each tumor section. For delimitation of the tumor regions core *vs*. periphery, 100 mm from the normal brain boundary was defined as periphery for all samples using a grid tool. The fraction of Ki67 positive against Ki67 negative tumor cells (Ki67 labeling index) was quantified in 5 microscopic high power fields (400 × magnification) in all animals in the study group. Detection of apoptotic cells was performed with the terminal deoxynucleotidyltransferase mediated nick endlabeling (TUNEL) assay according to the manufacturer's instructions (Roche Applied Bioscience, Mannheim, Germany) and apoptotic cells were quantified as previously described [79].

### NK cell purification and culture

Rat NK cells were purified by negative selection and cultured for 4-5 days with 1000 U/ml of IL-2 as previously described [17].

### TAMs and naïve microglia purification and culture

Human biopsy tissue and rat brain were dissected from the meninges and mechanically dissociated as previously described [6]. Rat homogenates were centrifuged and the pellet was suspended in 50 % isotonic Percoll (GE Healthcare, Brabant, Belgium), and then layered on the top of 70 % isotonic Percoll, and then 1XPBS was gently layered on the top. For human TAM isolation we used 40 % and 60 % isotonic Percoll gradient. After centrifugation at 1,200 × *g* for 35 min, microglia were collected at the interface between the 70 and 50 % for rat or 60 % and 40 % for human, isotonic Percoll. The purity of TAMs was always superior to 90 %. TAMs and naïve microglia were used directly for cytotoxicity assays against U87MG, HF66 and P3-30 or adjusted at 2.10^6^ cells/ml and cultured for 18 h or 96 h in complete RPMI-1640, or complete medium supplemented 100 ng/ml of rat IFN-γ (R&D Systems) (M1-like microglia) or GBM supernatant to get M2-like microglia.

### Flow cytometry staining

Rat cells were stained for surface antigens using: APC-Cy7–conjugated anti-CD45 (OX-1, Biolegend, DS Uithoorn, The Netherlands), Alexa 647-conjugated anti-NKRP1 (10/78, Biolegend), or anti-CD163 (ED2, AbD Serotec), APC-conjugated anti-CCR2 (475301, R&D Systems), PE-Cy7-conjugated anti-CD8 (OX-8, eBioscience), PerCP-efluor 710-conjugated anti-CD11b/c (OX-42, eBioscience), PE-conjugated anti-CD3 (G4.18, eBioscience), or anti-CD86 (24F, Biolegend), or anti-CCR7 (polyclonal, Abcam) coupled to goat anti-rabbit PE conjugated F(ab')_2_(Jackson Immunoresearch), Alexa Fluor 488-conjugated anti-CD62L (OX-85, Biolegend), or FITC-conjugated anti-CD4 (OX-38, Biolegend) or anti-MHC class II (HIS19, eBioscience). All samples were stained with LIVE/DEAD fixable Yellow (Invitrogen) in order to gate out dead cells. Then the cells were fixed and permeabilized using CytofixCytoperm solution (BD Biosciences) before staining with Alexa fluor 700-conjugated anti-CD68 (ED1, abD Serotec). Interferon-γ production was monitored by intracellular FACS staining after the addition of Golgi Plug (BD Biosciences) for 5h with FITC-conjugated anti-IFN-γ (DB-1, BD Pharmingen). Human isolated TAM were stained using FITC-conjugated CD11b (Immuno Tools), V450-conjugated CD45 (BD Biosciences) and PERCPCy5.5-conjugated CCR2 (Biolegend). Finally, before data acquisition using FACS Fortessa (BD Biosciences, Erembodegem, Belgium), nucleated cells were stained using Sytox blue (Invitrogen). Data analysis was performed using FACSDiva Software version 6.1.2 (BD Biosciences) and picture and histograms overlays were done on Flowjo (Tree Star).

### Cytotoxicity assays

The cytotoxicity assays were performed as previously described [17]. Briefly, NK cell cytotoxicity was determined after 4 days in culture with 1,000 U/ml of IL-2, and microglia cytotoxicity was performed with resting cells, differentiated microglia after activation with IFN-γ or as TAMs against the U87MG, HF66 and P3-30 target cells labeled with 5μM of CFSE (Sigma Aldrich), pre-incubated or not with 1mg/ml of PEGylated mAb9.2.27. Effector cells (E) were mixed with target cells (T) at E/T ratios of 2/1. After 18 h incubation at 37°C, cells were analyzed on a FACSCanto flow cytometer. To identify dead cells, 15 μM of the dead cell marker TO-PRO-3 was added. At least 2,500 target cells per sample were examined.

### Cytokine measurements

The levels of IL-1α, IL-6, IL-10, IFN-γ, and TNF-α in CSF of untreated control rats, athymic rats treated with mAb9.2.27 alone or NK+mAb9.2.27 and cytotoxicity assay culture supernatants from NK, NK+GBM cells or GBM cells only were measured by CBA (BD Biosciences) according to the manufacturer's protocol. The levels of MCP-1 and MCP-3 in plasma were performed with ELISA kit from eBioscience and USCN Life Science according to manufacturer's protocol.

### Statistical analysis

All results were expressed as mean± SEM from at least three independent experiments. A probability level of ≤0.05 was considered significant. We used One-Way (with Tukey's Multiple Comparison Test) or Two-Way (with Bonferroni *a priori* Post-hoc test) analysis of variance (ANOVA) with post-hoc analyses for comparisons of more than 2 groups or variables. Survival was analyzed using the Kaplan-Meier and the log rank test using Graphpad Prism 5.0 (Graphpad Software, La Jolla, CA).

## Supplementary Figures



## References

[1] Louis DN, Ohgaki H, Wiestler OD, Cavenee WK, Burger PC, Jouvet A, Scheithauer BW, Kleihues P (2007). The 2007 WHO classification of tumours of the central nervous system. Acta Neuropathol.

[2] Stupp R, Hegi ME, Mason WP, van den Bent MJ, Taphoorn MJ, Janzer RC, Ludwin SK, Allgeier A, Fisher B, Belanger K, Hau P, Brandes AA, Gijtenbeek J, Marosi C, Vecht CJ, Mokhtari K (2009). Effects of radiotherapy with concomitant and adjuvant temozolomide versus radiotherapy alone on survival in glioblastoma in a randomised phase III study: 5-year analysis of the EORTC-NCIC trial. Lancet Oncol.

[3] Freitas M, Malheiros S, Stavale JN, Biassi TP, Zamuner FT, de Souza Begnami M, Soares FA, Vettore AL (2013). Expression of cancer/testis antigens is correlated with improved survival in glioblastoma. Oncotarget.

[4] Mao XG, Hutt-Cabezas M, Orr BA, Weingart M, Taylor I, Rajan AK, Odia Y, Kahlert U, Maciaczyk J, Nikkhah G, Eberhart CG, Raabe EH (2013). LIN28A facilitates the transformation of human neural stem cells and promotes glioblastoma tumorigenesis through a pro-invasive genetic program. Oncotarget.

[5] Phillips JJ (2012). Novel therapeutic targets in the brain tumor microenvironment. Oncotarget.

[6] Svendsen A, Verhoeff JJ, Immervoll H, Brogger JC, Kmiecik J, Poli A, Netland IA, Prestegarden L, Planaguma J, Torsvik A, Kjersem AB, Sakariassen PO, Heggdal JI, Van Furth WR, Bjerkvig R, Lund-Johansen M (2011). Expression of the progenitor marker NG2/CSPG4 predicts poor survival and resistance to ionising radiation in glioblastoma. Acta Neuropathol.

[7] Behm FG, Smith FO, Raimondi SC, Pui CH, Bernstein ID (1996). Human homologue of the rat chondroitin sulfate proteoglycan, NG2, detected by monoclonal antibody 7.1, identifies childhood acute lymphoblastic leukemias with t(4;11)(q21;q23) or t(11;19)(q23;p13) and MLL gene rearrangements. Blood.

[8] Chekenya M, Enger PO, Thorsen F, Tysnes BB, Al-Sarraj S, Read TA, Furmanek T, Mahesparan R, Levine JM, Butt AM, Pilkington GJ, Bjerkvig R (2002). The glial precursor proteoglycan, NG2, is expressed on tumour neovasculature by vascular pericytes in human malignant brain tumours. Neuropathol Appl Neurobiol.

[9] Mauvieux L, Delabesse E, Bourquelot P, Radford-Weiss I, Bennaceur A, Flandrin G, Valensi F, MacIntyre EA (1999). NG2 expression in MLL rearranged acute myeloid leukaemia is restricted to monoblastic cases. Br J Haematol.

[10] Wang X, Osada T, Wang Y, Yu L, Sakakura K, Katayama A, McCarthy JB, Brufsky A, Chivukula M, Khoury T, Hsu DS, Barry WT, Lyerly HK, Clay TM, Ferrone S (2010). CSPG4 protein as a new target for the antibody-based immunotherapy of triple-negative breast cancer. J Natl Cancer Inst.

[11] Phillips HS, Kharbanda S, Chen R, Forrest WF, Soriano RH, Wu TD, Misra A, Nigro JM, Colman H, Soroceanu L, Williams PM, Modrusan Z, Feuerstein BG, Aldape K (2006). Molecular subclasses of high-grade glioma predict prognosis, delineate a pattern of disease progression, and resemble stages in neurogenesis. Cancer Cell.

[12] Chekenya M, Rooprai HK, Davies D, Levine JM, Butt AM, Pilkington GJ (1999). The NG2 chondroitin sulfate proteoglycan: role in malignant progression of human brain tumours. Int J Dev Neurosci.

[13] Wang X, Osada T, Wang Y, Yu L, Sakakura K, Katayama A, McCarthy JB, Brufsky A, Chivukula M, Khoury T, Hsu DS, Barry WT, Lyerly HK, Clay TM, Ferrone S (2010). CSPG4 protein as a new target for the antibody-based immunotherapy of triple-negative breast cancer. J Natl Cancer Inst.

[14] Marcus A, Eshhar Z (2011). Allogeneic adoptive cell transfer therapy as a potent universal treatment for cancer. Oncotarget.

[15] Rosenberg SA, Restifo NP, Yang JC, Morgan RA, Dudley ME (2008). Adoptive cell transfer: a clinical path to effective cancer immunotherapy. Nat Rev Cancer.

[16] Miller JS (2002). Biology of natural killer cells in cancer and infection. Cancer Invest.

[17] Castriconi R, Daga A, Dondero A, Zona G, Poliani PL, Melotti A, Griffero F, Marubbi D, Spaziante R, Bellora F, Moretta L, Moretta A, Corte G, Bottino C (2009). NK cells recognize and kill human glioblastoma cells with stem cell-like properties. J Immunol.

[18] Avril T, Vauleon E, Hamlat A, Saikali S, Etcheverry A, Delmas C, Diabira S, Mosser J, Quillien V (2012). Human glioblastoma stem-like cells are more sensitive to allogeneic NK and T cell-mediated killing compared with serum-cultured glioblastoma cells. Brain Pathol.

[19] Kalinski P, Giermasz A, Nakamura Y, Basse P, Storkus WJ, Kirkwood JM, Mailliard RB (2005). Helper role of NK cells during the induction of anticancer responses by dendritic cells. Mol Immunol.

[20] Doherty PC, Allan JE (1987). Anti-asialo GM1 eliminates both inflammatory process and cytotoxic T-cell function in the lymphocytic choriomeningitis adoptive transfer model. Cell Immunol.

[21] Kelly JM, Darcy PK, Markby JL, Godfrey DI, Takeda K, Yagita H, Smyth MJ (2002). Induction of tumor-specific T cell memory by NK cell-mediated tumor rejection. Nat Immunol.

[22] Heusinkveld M, van der Burg SH (2011). Identification and manipulation of tumor associated macrophages in human cancers. J Transl Med.

[23] O'Sullivan T, Saddawi-Konefka R, Vermi W, Koebel CM, Arthur C, White JM, Uppaluri R, Andrews DM, Ngiow SF, Teng MW, Smyth MJ, Schreiber RD, Bui JD (2012). Cancer immunoediting by the innate immune system in the absence of adaptive immunity. J Exp Med.

[24] Morantz RA, Wood GW, Foster M, Clark M, Gollahon K (1979). Macrophages in experimental and human brain tumors. Part 2: studies of the macrophage content of human brain tumors. J Neurosurg.

[25] Kmiecik J, Poli A, Brons NHC, Waha A, Eided GE, Enger PO, Zimmer J, Chekenya (2013). Elevated CD3 + and CD8 + tumor–infiltrating immune cells correlates with prolonged survival in glioblastoma patients despite integrated immunosuppressive mechanisms in the tumor microenvironment and at the systemic level. Journal of Neuroimmunology.

[26] Romagne F, Vivier E (2011). Natural killer cell-based therapies. F1000 medicine reports.

[27] Zhang Z, Artelt M, Burnet M, Trautmann K, Schluesener HJ (2006). Early infiltration of CD8+ macrophages/microglia to lesions of rat traumatic brain injury. Neuroscience.

[28] Wang J, Miletic H, Sakariassen PO, Huszthy PC, Jacobsen H, Brekka N, Li X, Zhao P, Mork S, Chekenya M, Bjerkvig R, Enger PO (2009). A reproducible brain tumour model established from human glioblastoma biopsies. BMC cancer.

[29] Yang I, Han SJ, Kaur G, Crane C, Parsa AT (2010). The role of microglia in central nervous system immunity and glioma immunology. J Clin Neurosci.

[30] Rolny C, Mazzone M, Tugues S, Laoui D, Johansson I, Coulon C, Squadrito ML, Segura I, Li X, Knevels E, Costa S, Vinckier S, Dresselaer T, Akerud P, De Mol M, Salomaki H (2011). HRG inhibits tumor growth and metastasis by inducing macrophage polarization and vessel normalization through downregulation of PlGF. Cancer Cell.

[31] Kees T, Lohr J, Noack J, Mora R, Gdynia G, Todt G, Ernst A, Radlwimmer B, Falk CS, Herold-Mende C, Regnier-Vigouroux A (2012). Microglia isolated from patients with glioma gain antitumor activities on poly (I:C) stimulation. Neuro-oncology.

[32] Munoz DM, Guha A (2011). Mouse models to interrogate the implications of the differentiation status in the ontogeny of gliomas. Oncotarget.

[33] Sakariassen PO, Prestegarden L, Wang J, Skaftnesmo KO, Mahesparan R, Molthoff C, Sminia P, Sundlisaeter E, Misra A, Tysnes BB, Chekenya M, Peters H, Lende G, Kalland KH, Oyan AM, Petersen K (2006). Angiogenesis-independent tumor growth mediated by stem-like cancer cells. Proc Natl Acad Sci U S A.

[34] Smith MM, Thompson JE, Castillo M, Cush S, Mukherji SK, Miller CH, Quattrocchi KB (1996). MR of recurrent high-grade astrocytomas after intralesional immunotherapy. AJNR American journal of neuroradiology.

[35] Floeth FW, Aulich A, Langen KJ, Burger KJ, Bock WJ, Weber F (2001). MR imaging and single-photon emission CT findings after gene therapy for human glioblastoma. AJNR American journal of neuroradiology.

[36] Abbott NJ (2004). Evidence for bulk flow of brain interstitial fluid: significance for physiology and pathology. Neurochemistry international.

[37] Matheus MG, Castillo M, Ewend M, Smith JK, Knock L, Cush S, Morris DE (2004). CT and MR imaging after placement of the GliaSite radiation therapy system to treat brain tumor: initial experience. AJNR American journal of neuroradiology.

[38] van den Bent MJ, Vogelbaum MA, Wen PY, Macdonald DR, Chang SM (2009). End point assessment in gliomas: novel treatments limit usefulness of classical Macdonald's Criteria. J Clin Oncol.

[39] Byrne KT, Turk MJ (2011). New perspectives on the role of vitiligo in immune responses to melanoma. Oncotarget.

[40] Aloisi F, Ria F, Adorini L (2000). Regulation of T-cell responses by CNS antigen-presenting cells: different roles for microglia and astrocytes. Immunol Today.

[41] Charles N, Holland EC (2010). The perivascular niche microenvironment in brain tumor progression. Cell cycle.

[42] Zhang J, Sarkar S, Cua R, Zhou Y, Hader W, Yong VW (2012). A dialog between glioma and microglia that promotes tumor invasiveness through the CCL2/CCR2/interleukin-6 axis. Carcinogenesis.

[43] Van Rooijen N, Sanders A (1994). Liposome mediated depletion of macrophages: mechanism of action, preparation of liposomes and applications. J Immunol Methods.

[44] Rabinovich GA, Riera CM, Iribarren P (1999). Granulocyte-macrophage colony-stimulating factor protects dendritic cells from liposome-encapsulated dichloromethylene diphosphonate-induced apoptosis through a Bcl-2-mediated pathway. Eur J Immunol.

[45] Brenan M, Puklavec M (1992). The MRC OX-62 antigen: a useful marker in the purification of rat veiled cells with the biochemical properties of an integrin. J Exp Med.

[46] Brenan M, Rees DJ (2000). Sequence analysis or rat integrin alphaE1 and alphaE2 subunits: tissue expression reveals phenotypic similarities between intraepithelial lymphocytes and dendritic cells in lymph. Eur J Immunol.

[47] Voisine C, Hubert FX, Trinite B, Heslan M, Josien R (2002). Two phenotypically distinct subsets of spleen dendritic cells in rats exhibit different cytokine production and T cell stimulatory activity. J Immunol.

[48] Guo K, Tang JP, Jie L, Al-Aidaroos AQ, Hong CW, Tan CP, Park JE, Varghese L, Feng Z, Zhou J, Chng WJ, Zeng Q (2012). Engineering the first chimeric antibody in targeting intracellular PRL-3 oncoprotein for cancer therapy in mice. Oncotarget.

[49] Wang R, Chadalavada K, Wilshire J, Kowalik U, Hovinga KE, Geber A, Fligelman B, Leversha M, Brennan C, Tabar V (2010). Glioblastoma stem-like cells give rise to tumour endothelium. Nature.

[50] Ricci-Vitiani L, Pallini R, Biffoni M, Todaro M, Invernici G, Cenci T, Maira G, Parati EA, Stassi G, Larocca LM, De Maria R (2010). Tumour vascularization via endothelial differentiation of glioblastoma stem-like cells. Nature.

[51] Rodriguez FJ, Orr BA, Ligon KL, Eberhart CG (2012). Neoplastic cells are a rare component in human glioblastoma microvasculature. Oncotarget.

[52] Fukushi J, Makagiansar IT, Stallcup WB (2004). NG2 proteoglycan promotes endothelial cell motility and angiogenesis via engagement of galectin-3 and alpha3beta1 integrin. Mol Biol Cell.

[53] Ozerdem U, Stallcup WB (2004). Pathological angiogenesis is reduced by targeting pericytes via the NG2 proteoglycan. Angiogenesis.

[54] Teeling JL, Carare RO, Glennie MJ, Perry VH (2012). Intracerebral immune complex formation induces inflammation in the brain that depends on Fc receptor interaction. Acta Neuropathol.

[55] Sutter A, Hekmat A, Luckenbach GA (1991). Antibody-mediated tumor cytotoxicity of microglia. Pathobiology.

[56] Sampson JH, Crotty LE, Lee S, Archer GE, Ashley DM, Wikstrand CJ, Hale LP, Small C, Dranoff G, Friedman AH, Friedman HS, Bigner DD (2000). Unarmed, tumor-specific monoclonal antibody effectively treats brain tumors. Proc Natl Acad Sci U S A.

[57] Okun E, Mattson MP, Arumugam TV (2010). Involvement of Fc receptors in disorders of the central nervous system. Neuromolecular Med.

[58] Nogueira L, Ruiz-Ontanon P, Vazquez-Barquero A, Moris F, Fernandez-Luna JL (2011). The NFkappaB pathway: a therapeutic target in glioblastoma. Oncotarget.

[59] Duluc D, Corvaisier M, Blanchard S, Catala L, Descamps P, Gamelin E, Ponsoda S, Delneste Y, Hebbar M, Jeannin P (2009). Interferon-gamma reverses the immunosuppressive and protumoral properties and prevents the generation of human tumor-associated macrophages. Int J Cancer.

[60] Suzumura A, Mezitis SG, Gonatas NK, Silberberg DH (1987). MHC antigen expression on bulk isolated macrophage-microglia from newborn mouse brain: induction of Ia antigen expression by gamma-interferon. J Neuroimmunol.

[61] Williams K, Ulvestad E, Antel JP (1994). B7/BB-1 antigen expression on adult human microglia studied in vitro and in situ. Eur J Immunol.

[62] Chan A, Magnus T, Gold R (2001). Phagocytosis of apoptotic inflammatory cells by microglia and modulation by different cytokines: mechanism for removal of apoptotic cells in the inflamed nervous system. Glia.

[63] Xiao BG, Bai XF, Zhang GX, Hojeberg B, Link H (1996). Shift from anti- to proinflammatory cytokine profiles in microglia through LPS- or IFN-gamma-mediated pathways. Neuroreport.

[64] Lefkowitz DL, Lefkowitz SS (2008). Microglia and myeloperoxidase: a deadly partnership in neurodegenerative disease. Free Radic Biol Med.

[65] Butovsky O, Ziv Y, Schwartz A, Landa G, Talpalar AE, Pluchino S, Martino G, Schwartz M (2006). Microglia activated by IL-4 or IFN-gamma differentially induce neurogenesis and oligodendrogenesis from adult stem/progenitor cells. Molecular and cellular neurosciences.

[66] Butovsky O, Talpalar AE, Ben-Yaakov K, Schwartz M (2005). Activation of microglia by aggregated beta-amyloid or lipopolysaccharide impairs MHC-II expression and renders them cytotoxic whereas IFN-gamma and IL-4 render them protective. Molecular and cellular neurosciences.

[67] Bellora F, Castriconi R, Dondero A, Reggiardo G, Moretta L, Mantovani A, Moretta A, Bottino C (2010). The interaction of human natural killer cells with either unpolarized or polarized macrophages results in different functional outcomes. Proc Natl Acad Sci U S A.

[68] Rolstad B (2001). The athymic nude rat: an animal experimental model to reveal novel aspects of innate immune responses?. Immunological reviews.

[69] Luo XG, Ding JQ, Chen SD (2010). Microglia in the aging brain: relevance to neurodegeneration. Mol Neurodegener.

[70] Dheen ST, Kaur C, Ling EA (2007). Microglial activation and its implications in the brain diseases. Curr Med Chem.

[71] Song ES, Young K, Sears DW (1990). Rat and human natural killers exhibit contrasting immunoglobulin G subclass specificities in antibody-dependent cellular cytotoxicity reflecting differences in their Fc receptors (Fc gamma R). J Leukoc Biol.

[72] Jones JF, Segal DM (1980). Antibody-dependent cell-mediated cytolysis (ADCC) with antibody-coated effectors: new methods for enhancing antibody binding and cytolysis. J Immunol.

[73] Festing MF, May D, Connors TA, Lovell D, Sparrow S (1978). An athymic nude mutation in the rat. Nature.

[74] Vos JG, Kreeftenberg JG, Kruijt BC, Kruizinga W, Steerenberg P (1980). The athymic nude rat. II. Immunological characteristics. Clin Immunol Immunopathol.

[75] Cook JL, Ikle DN, Routes BA (1995). Natural killer cell ontogeny in the athymic rat. Relationship between functional maturation and acquired resistance to E1A oncogene-expressing sarcoma cells. J Immunol.

[76] Wang J, Miletic H, Sakariassen PO, Huszthy PC, Jacobsen H, Brekka N, Li X, Zhao P, Mork S, Chekenya M, Bjerkvig R, Enger PO (2009). A reproducible brain tumour model established from human glioblastoma biopsies. BMC cancer.

[77] Cunningham CL, Martinez-Cerdeno V, Noctor SC (2013). Microglia regulate the number of neural precursor cells in the developing cerebral cortex. The Journal of neuroscience : the official journal of the Society for Neuroscience.

[78] Drabek T, Janata A, Jackson EK, End B, Stezoski J, Vagni VA, Janesko-Feldman K, Wilson CD, van Rooijen N, Tisherman SA, Kochanek PM (2012). Microglial depletion using intrahippocampal injection of liposome-encapsulated clodronate in prolonged hypothermic cardiac arrest in rats. Resuscitation.

[79] Wang J, Svendsen A, Kmiecik J, Immervoll H, Skaftnesmo KO, Planaguma J, Reed RK, Bjerkvig R, Miletic H, Enger PO, Rygh CB, Chekenya M (2011). Targeting the NG2/CSPG4 proteoglycan retards tumour growth and angiogenesis in preclinical models of GBM and melanoma. PLoS One.

[80] Lamprecht MR, Sabatini DM, Carpenter AE (2007). CellProfiler: free, versatile software for automated biological image analysis. BioTechniques.

