# Supplementary material for: Targeting glioblastoma with NK cells and mAb against NG2/CSPG4 prolongs animal survival
Source: Oncotarget. 2013 Sep 9;4(9):1527–46. doi: 10.18632/oncotarget.1291 (PMC3824525; doi:10.18632/oncotarget.1291)
Supplement: Supplementary file 1 [file oncotarget-04-1527-s001.pdf]

Targeting glioblastoma with NK cells and mAb against NG2/*CSPG4* prolongs animal survival – Poli et al

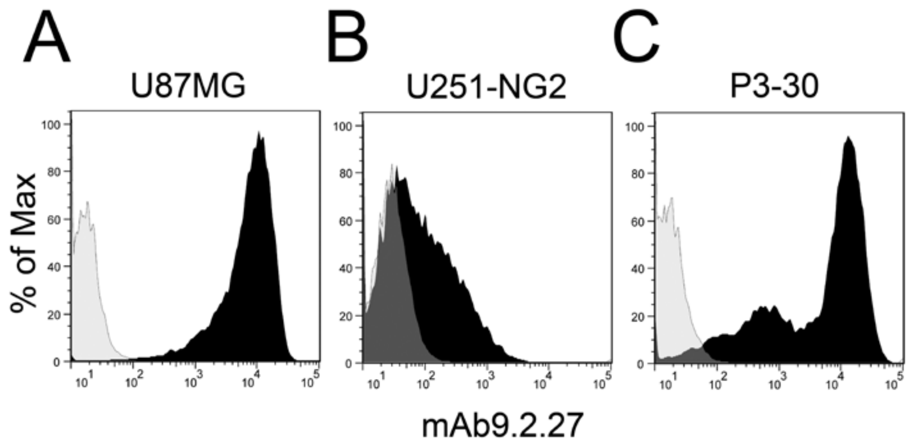

Supplementary Figure 1: Level of NG2 recognition by mAb9.2.27 on U87MG, U251-NG2 and P3-30. (A) Representative flow cytometric histograms showing NG2 expression in U87MG, **(B)** U251-NG2 and **(C)** P3-30 GBM in vitro, labeled with mAb9.2.27 (filled black histograms) or mouse IgG2a isotype control (tinted grey histograms). % NG2 expression represents mean ±SEM, from 3 independent experiments.

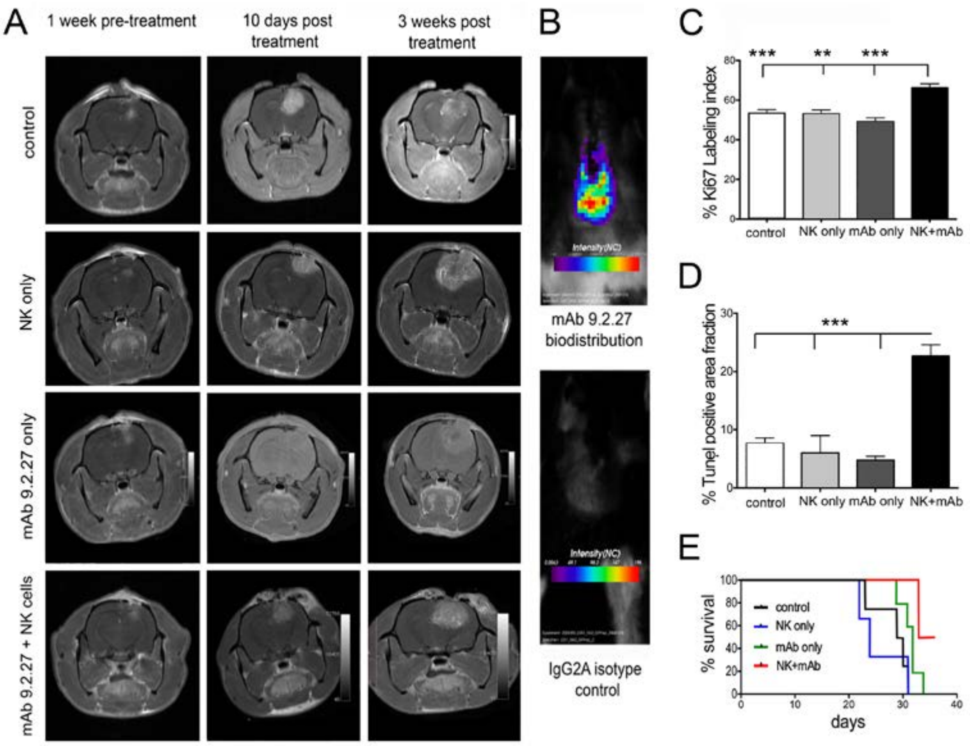

Supplementary Figure 2: mAb9.2.27 localizes to tumor bed, diminishes vascular permeability and prolongs survival in U251-NG2 tumors. (A) Axial post-contrast T1-weighted images of nude rats bearing NG2 overexpressing U251-NG2 tumors, vehicle controls, NK-cell monotherapy, mAb9.2.27 monotherapy, and combination NK+mAb9.2.27 treatment**. (B)** Bio-distribution of near infrared conjugated mAb9.2.27 (upper panel) and IgG2a isotype control (lower panel) 6 days post convection enhanced delivery by osmotic pumps. **(C)** Quantification of tumor proliferation by Ki67 labeling index. **(D)** Quantification of cell death (area fraction TUNEL positive apoptotic/necrotic cells). Data represent mean ±SEM of all tumors in the groups, ***p<0.0001, **p<0.01. **(E)** Kaplan–Meier survival curves showing % survival. The experiment was terminated when all control groups had been sacrificed due to tumor related symptoms. At that point 50% of the combination NK+mAb9.2.27 treated animals were alive.

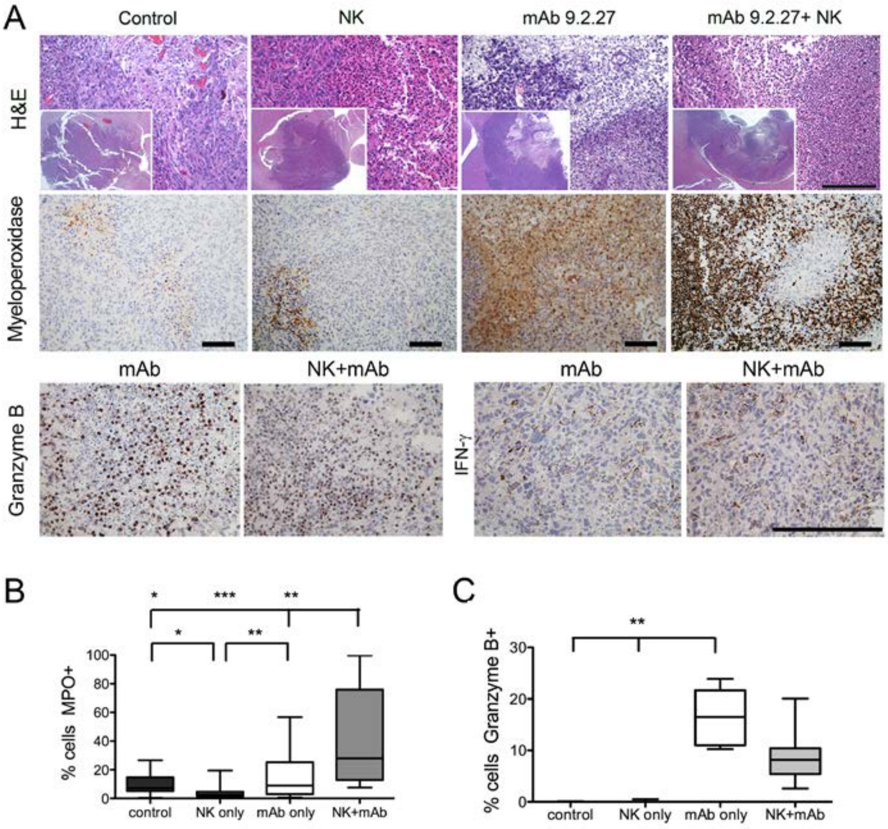

Supplementary Figure 3: Confirmation of a tropism for cytotoxic cells in combination treated from U251-NG2 bearing animals. (A) H&E images of the U251-NG2 control, monotherapy NK cells, mAb9.2.27 and

NK+mAb9.2.27 combination treated tumors show immune cells and varying necrosis, largest in mAb9.2.27 and combination treated tumors (Scale bar 200μm, Magnification 100X). Inserts are overview images, magnification, 25 X. Abundant MPO expressing cells present in the NK+mAb9.2.27 combination treated tumors compared to control and monotherapy mAb9.2.27 as well as NK monotherapy. Granzyme B and IFN-γ staining were visible only in mAb9.2.27 monotherapy and combined treatment. Quantification of % cells positive **(B)** MPO expressing cells and **(C)** Granzyme B positive cells in the control, monotherapy mAb9.2.27, NK monotherapy and NK+mAb9.2.27 combination treated tumors. Data in **(B)** and **(C)** represent mean ±SEM from all tumors in the groups, $^{**}p<0.001$, $^{***}p<0.0001$.

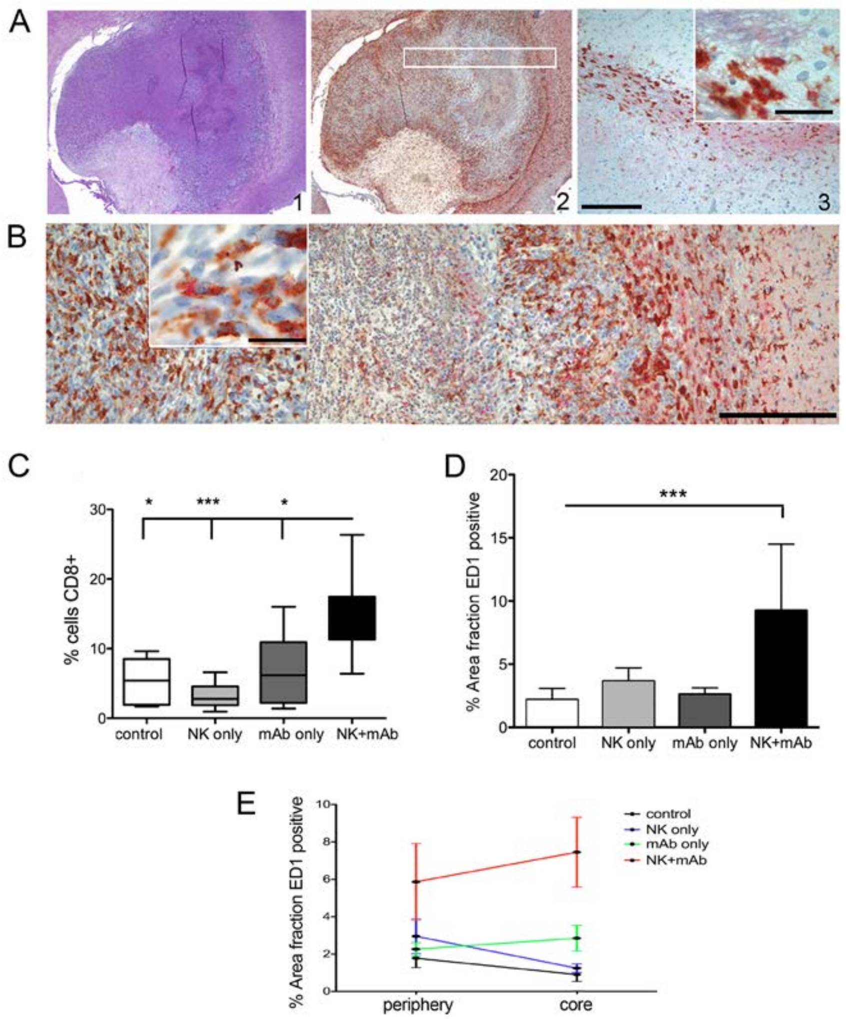

Supplementary Figure 4: **Differential macrophage/microglia phenotypes in response to treatment.** (**A1)** H&E image of representative NK+mAb9.2.27 combination treated tumor, (**A2)** Overview image of same tumor double stained for CD8 (brown) and ED1 (red) showing abundant tumor infiltration by double CD8/ED1 positive cells. Fibrotic, old necrosis as well as fresh necrotic region (blue/grey) in the tumor core, **(A3)** Infiltration of CD8/ED1 double positive cells (insert, scale bar 100 μm, magnification 1000 X) via white matter tracts in contralateral hemisphere towards the tumor, **(B)** Transition images from a region of interest (white bar in **A2**) showing infiltration of double CD8/ED1 positive cells CD8 (brown) and ED1 (red) from normal brain towards the tumor core, (insert, scale bar 100μm, magnification 1000X). Note region of fresh necrosis filled with immune cells (Scale bar 200μm, Magnification 200X) Quantification of % area fraction **(C)** CD8, **(D)** ED1 positive cells and (**E**) ED1 positive cells in tumor core versus periphery in the control, monotherapy mAb9.2.27, NK monotherapy and NK+mAb9.2.27 combination treated tumors. Data in **(C)** and **(D)** and (**E**) represent mean ±SEM from all tumors in the groups, ***p<0.0001, *p<0.01.

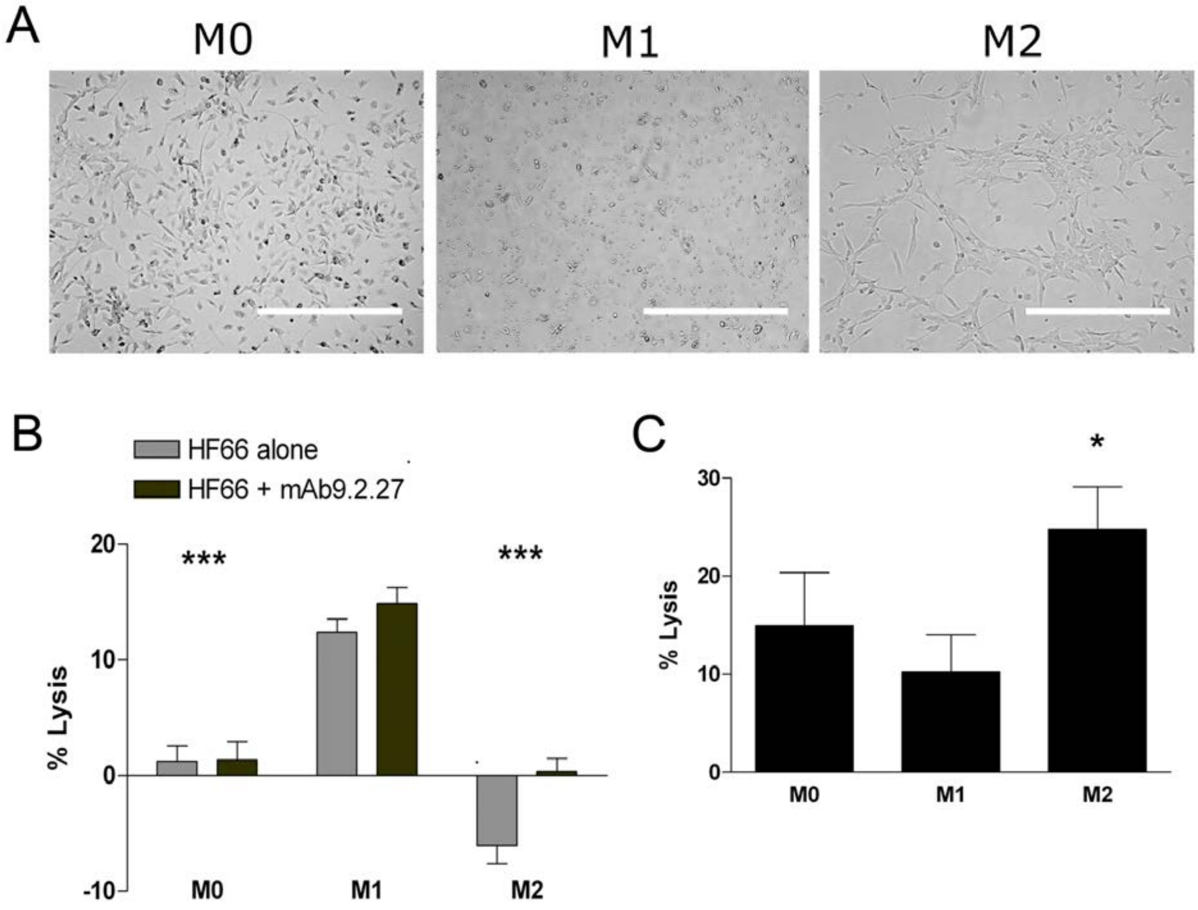

Supplementary Figure 5. Microglia with M0, M1 or M2-like phenotype show differential cytotoxic activity against HF66 and susceptibility to NK-cell lysis.

(A) Microglia were purified from brain cells by Percoll gradient and put in culture with medium only, medium supplemented with 100 ng/ml IFN-γ or HF66 conditioned medium for 5 days in order to differentiate them into M0, M1 and M2-like phenotype, respectively. (B) These cells served as effectors for cytotoxicity assay against HF66 pre-incubated or not with PEGmAb9.2.27 or (C) as targets for cytotoxicity assay with activated NK-cells as effectors. Data are plotted as mean ±SEM from three independent experiments. ***$p<0.0001$, *$p<0.01$.

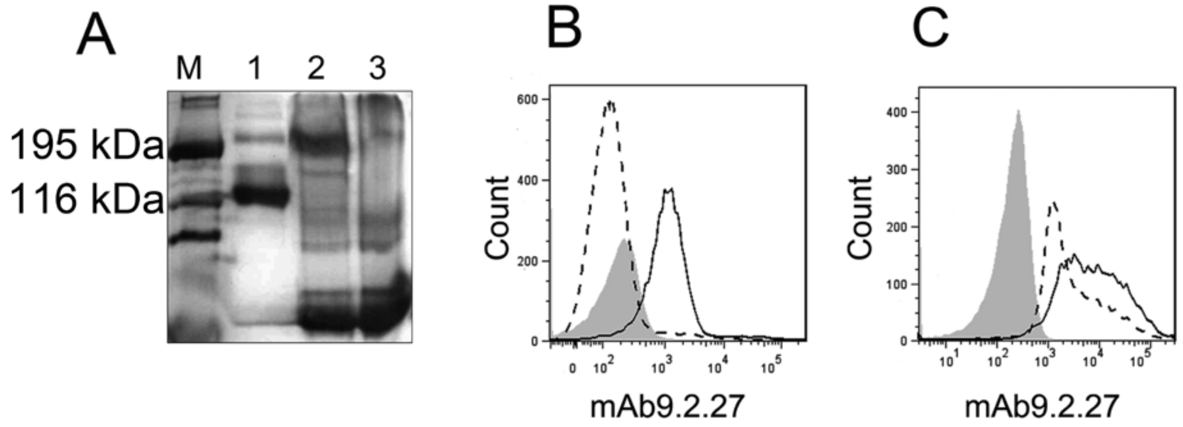

Supplementary Figure 6: **Fc Receptors of NK and microglial cells have a better affinity for PEGylated mAb9.2.27. (A)** The PEGylation of the mAb9.2.27 was verified by SDS-PAGE and the gel stained by Comassie blue (lane M: molecular weight, lane 1: mAb9.2.27 alone, lane 2: PEGmAb9.2.27 and lane 3: 24 % PEG solution). **(B)** The purified NK cells and **(C)** microglia were incubated at 4 °C for 30 min with PEGylated (straight line), unPEGylated (dotted line) mAb9.2.27 or IgG2a isotype control alone (filled line), and incubated with PE-conjugated anti-mouse antibody to determine the affinity of cells for mAb9.2.27 or isotype control mAb. These data represent at least 3 independent experiments.

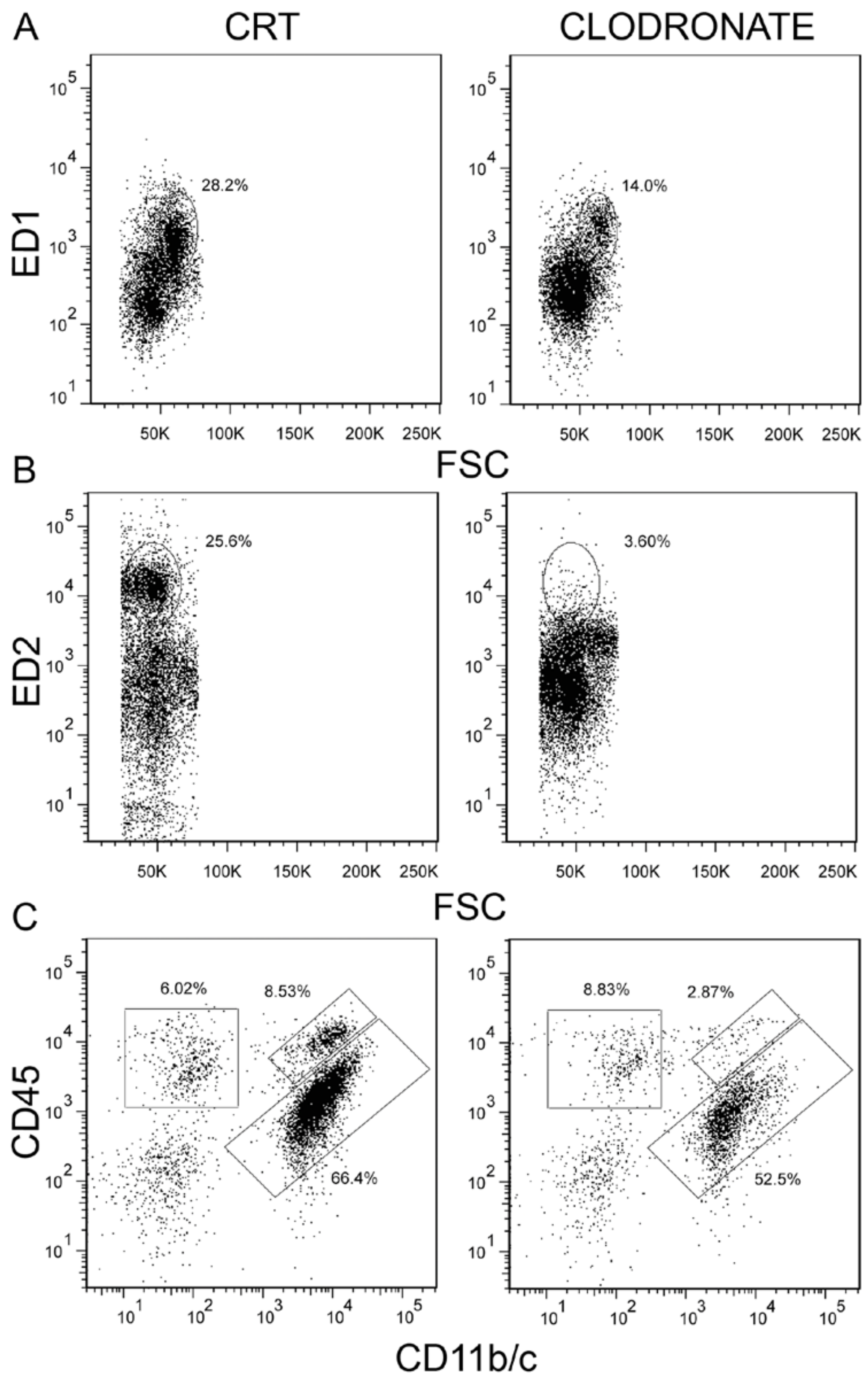

Supplementary Figure 7: **Liposome-encapsulated clodronate depleted ED1 and ED2 macrophages from the spleen of tumor bearing rats, but not microglia from the brain.** Depletion of macrophages was verified by flow cytometry staining of splenocytes, 3 days after first injection, for ED1 **(A)** and ED2 **(B)**. Dot plots from a representative animals showing ED1 **(A)** or ED2 **(B)** against cell size (FSC), after i.p injection of

PBS/liposome as control **(CRT : left panel)** and i.p injection of encapsulated clodronate **(CLODRONATE : right panel)**. Comparison of cellular composition of resident lymphocytes, $CD45^{high}CD11b/c$, macrophages, $CD45^{high}CD11b/c^{+}$ and microglia $CD45^{low}CD11b/c^{+}$, 3 days post i.p injection of liposome-encapsulated clodronate, showing the depletion of macrophages infiltrating the brain **(C).**
